# The Genome Response to Artificial Selection: A Case Study in Dairy Cattle

**DOI:** 10.1371/journal.pone.0006595

**Published:** 2009-08-12

**Authors:** Laurence Flori, Sébastien Fritz, Florence Jaffrézic, Mekki Boussaha, Ivo Gut, Simon Heath, Jean-Louis Foulley, Mathieu Gautier

**Affiliations:** 1 INRA, UMR de Génétique Animale et Biologie Intégrative, Jouy-en-Josas, France; 2 Union Nationale des Coopératives agricoles d'Elevage et d'Insémination Animale, Paris, France; 3 Centre National de Genotypage, Institut de Génomique, Commissariat à l'Energie Atomique, Evry, France; University of Utah, United States of America

## Abstract

Dairy cattle breeds have been subjected over the last fifty years to intense artificial selection towards improvement of milk production traits. In this study, we performed a whole genome scan for differentiation using 42,486 SNPs in the three major French dairy cattle breeds (Holstein, Normande and Montbéliarde) to identify the main physiological pathways and regions which were affected by this selection. After analyzing the population structure, we estimated *F_ST_* within and across the three breeds for each SNP under a pure drift model. We further considered two different strategies to evaluate the effect of selection at the genome level. First, smoothing *F_ST_* values over each chromosome with a local variable bandwidth kernel estimator allowed identifying 13 highly significant regions subjected to strong and/or recent positive selection. Some of them contained genes within which causal variants with strong effect on milk production traits (GHR) or coloration (MC1R) have already been reported. To go further in the interpretation of the observed signatures of selection we subsequently concentrated on the annotation of differentiated genes defined according to the *F_ST_* value of SNPs localized close or within them. To that end we performed a comprehensive network analysis which suggested a central role of somatotropic and gonadotropic axes in the response to selection. Altogether, these observations shed light on the antagonism, at the genome level, between milk production and reproduction traits in highly producing dairy cows.

## Introduction

As for other domestic animals, both natural and artificial selection have resulted over a short period of time in a broad phenotypic variety and in genetic differentiation of numerous different cattle breeds. This recent history provides a unique opportunity for the identification of loci subjected to adaptive selection. Following domestication, about ∼10,000 years ago, early breeders might have imposed a so-called “unconscious” selection “which results from every one trying to possess and breed from the best individual animals” [1]. Following innovative farmers such as Robert Bakewell (1725–1795), selection recently became more methodical in industrialized countries, in particular with the opening of the first herd-books which strictly defined the breed standards. Subsequent advances in theoretical understanding of the inheritance of quantitative traits and their application to genetic improvement have made it possible to reach a high degree of specialization in several breeds for the last fifty years. A spectacular example of success of such genetic improvement programmes is offered by dairy cattle breeds [2].

Currently, more than 95% of the cows milked in France belong to Holstein (HOL), Normande (NOR) or Montbéliarde (MON) breeds. The herd-book of these three different breeds were created in 1922, 1883 and 1872 respectively using individuals originated from distant areas (North of Europe, North-western France and Mid-eastern France). Since the middle of the twentieth century, these three breeds have been subjected to strong artificial selection mainly oriented towards an improvement of dairy abilities. Nonetheless, because of varying local breeder objectives and herding systems, these breeds displayed some differences in most of their milk production traits (quantity and quality of milk) and on other morphological characteristics (color, stature) as broadly summarized in Table 1. On the other hand, although highly effective, enhancement of milk production abilities in highly producing dairy cows has also been accompanied by a marked decline for other functional traits such as reproductive performances [3], [4]. For instance, negative genetic correlations (from −0.30 to −0.50) between milk quantity and Artificial Insemination (AI) success have been reported in a large scale study performed in HOL, NOR and MON [5].

**Table 1:** General characteristics of the three breeds studied (http://www.brg.prd.fr).

Breed	Census Population Size	Lactation Length (in days)	Milk Yield (in L)	Fat Percentage (‰)	Protein Percentage(‰)	Male Height (in cm)/Weight (in kg)	Female Height (in cm)/Weight (in kg)
N	1,799,200	317	7441	38.8	32.5	150/1100	144/700
NOR	2,106,000	316	6595	44.2	36.0	155/1100	142/800
HOL	11,535,378	331	8628	40.9	31.6	165/1100	143/700

Data were collected in year 2005.

The advent of high throughput and cost-effective genotyping techniques allows evaluating the response to these various selective pressures at the genome level. For instance, comparing allele frequencies or differentiation among different breeds is straightforward to identify footprints of selection which are characterized by an unexpectedly high level of divergence, relatively to the neutral hypothesis [6], [7]. Recently, Hayes *et al.* [8] proved the efficiency of such an approach with the analysis of 9,323 SNPs genotyped on samples from a dairy and a beef cattle breed. Most beneficial mutations are likely to be quite old relatively to the very recent breed formation times, as exemplified by the DGAT1 K232A mutations underlying a QTL with major effect on dairy traits and still segregating in several dairy cattle breeds [9]–[12]. A variant selected in one breed is thus expected to exhibit frequency differences when compared to other breeds in which it might have only been subjected to genetic drift. In addition, these differences are expected to be the most extreme for variants initially at low frequency and with strong effect in some of the populations considered. Alternatively, even if similar selection goals might have driven to fixation the same variant in all the breeds compared, different SNP alleles might still be associated to it at more distant loci. Indeed, Linkage Disequilibrium (LD) across breeds was shown to only persist over few kb [13], [14] which is still below the available density of current SNP chips. Hence, analyzing differentiation among breeds with similar breeding objectives is expected to be efficient in identifying loci which were early selected while providing results easier to interpret in the light of their shared selective pressure.

The goal of this study was to perform a genome scan for SNP differentiation, by considering 42,846 SNPs genotyped in HOL, MON and NOR dairy cattle breeds, to identify the main regions affected by the strong and recent artificial selection they have been subjected to. To that end we applied and extended previously proposed approaches [6], [7]. Finally, based on a list of several genes displaying high evidence of selection, we further carried out a detailed and comprehensive functional and network analysis, under a systems biology framework, to characterize the main targeted physiological pathways.

## Results and Discussion

### Population structure and distribution of F_ST_ among SNPs and populations

**Figure 1:**
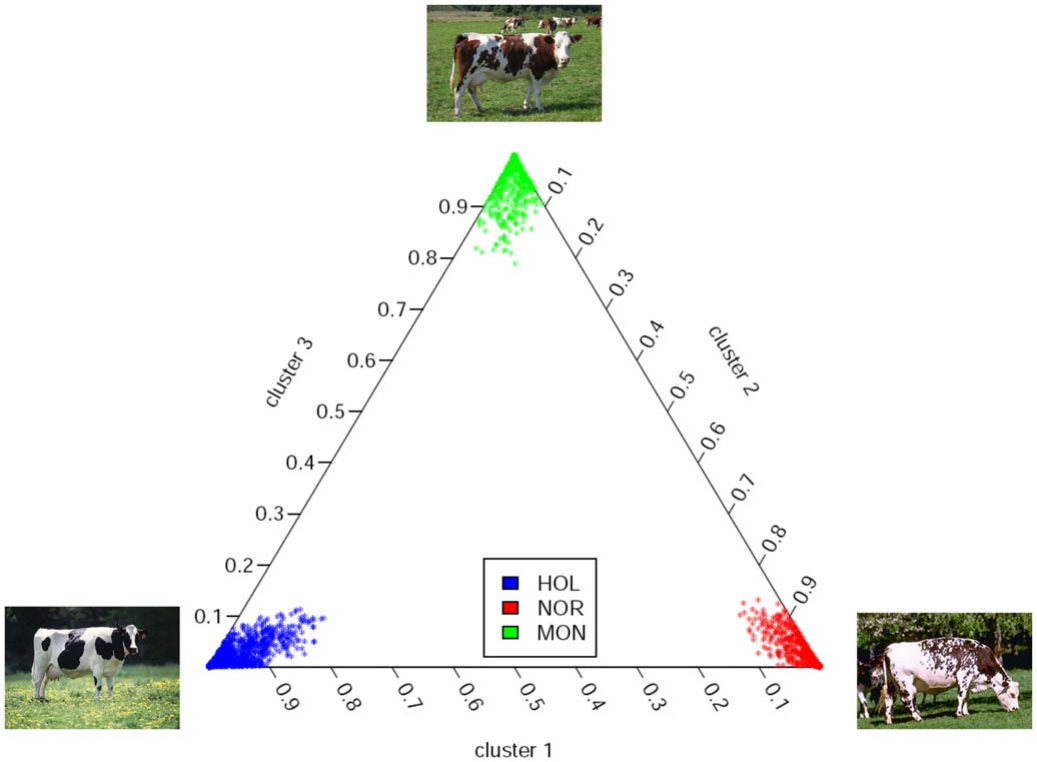
Population structure. The triangle plot represents the estimated membership of each 2803 bulls in each of the 3 assumed clusters. Each bull is represented by a point colored according to its breed of origin.

As expected from the recent breed history, unsupervised clustering of the 2803 bulls belonging to HOL, MON and NOR breeds (Table S1) highlighted their nearly complete genetic isolation by confirming an almost complete absence of admixture (Figure 1). Indeed, when considering K = 3 unknown parental populations, each cluster could be unambiguously assigned to a breed. The average (median) proportions of HOL bulls membership was respectively 96.0% (97.8%) in cluster 1, 96.1% (97.7%) for NOR bulls in cluster 2 and 95.7% (97.0%) for MON bulls in the cluster 3. In addition, both the average values of cluster differentiation (0.134 for “HOL” cluster, 0.125 for the “NOR” cluster 2 and 0.155 for the “MON” cluster 3) and the net nucleotide distances among cluster pairs (from 0.046 between the first and the second cluster to 0.055 for the first and third cluster) suggested similar level of differentiation among the underlying three breeds. We thus estimated *F_ST_* under the pure-drift model proposed by Nicholson *et al.* [15] for 42,286 SNPs both within and across the three breeds (Table S2). Note that under this model, population-specific *F_ST_* are computed relatively to the ancestral population and their inverse might thus be interpreted as an effective size of bottleneck (see Material and Methods). Overall, the average *F_ST_* across breeds was equal to 0.0709 (from 0.0576 for BTA27 to 0.0840 for BTA05) and population-specific *F_ST_* were all close to this average value (0.0696 for MON, 0.0688 for NOR and 0.0743 for HOL). The slightly higher HOL average *F_ST_* might reflect the more distant geographic origin (Northern Europe) of animals from which this breed originates, compared to NOR (North-Western France) and MON ones (Eastern France).

As mentioned above, such levels of population differentiation would be expected from a common ancestral population with a bottleneck starting 25 generations ago (∼150 years ago if we assume a generation time of about 6 years in dairy cattle) and a constant (haploid) effective population size varying from 340 (in HOL) to 360 (in NOR). These effective population size estimates appeared somewhat upwardly biased when compared to those derived from the extent of LD [14]. Likewise, simulations under a simple pure-drift model required a marked decrease in simulated population sizes to give a good fit with observed data (see Methods). Although the SNP ascertainment scheme chosen in our study could explain such apparent discrepancies, the main explanation might rather be related to the downward bias introduced by the methods of moments' estimators [15] since it imposes, in particular, the ancestral allele frequency estimates to be within the range of the current populations' ones. Simple simulations under the inference model confirmed that the more the populations considered are differentiated the higher the bias (data not shown). Similarly, the average *F_ST_* across populations was substantially lower than the one (0.0710 against 0.103) computed when using the Weir and Cockerham estimator [6], [16], [17], the estimates of the individual SNP *F_ST_* being nevertheless highly correlated (*r* = 0.961) between the two methods. This suggested that the resulting classification of SNPs is rather insensitive to bias introduced by our estimation procedure, the main advantage of this latter being the simple computation of population-specific estimates. Therefore, providing populations are well but not too much differentiated (low admixture), this procedure might be thought of as a straightforward way to compare allele frequencies across several populations.

**Figure 2:**
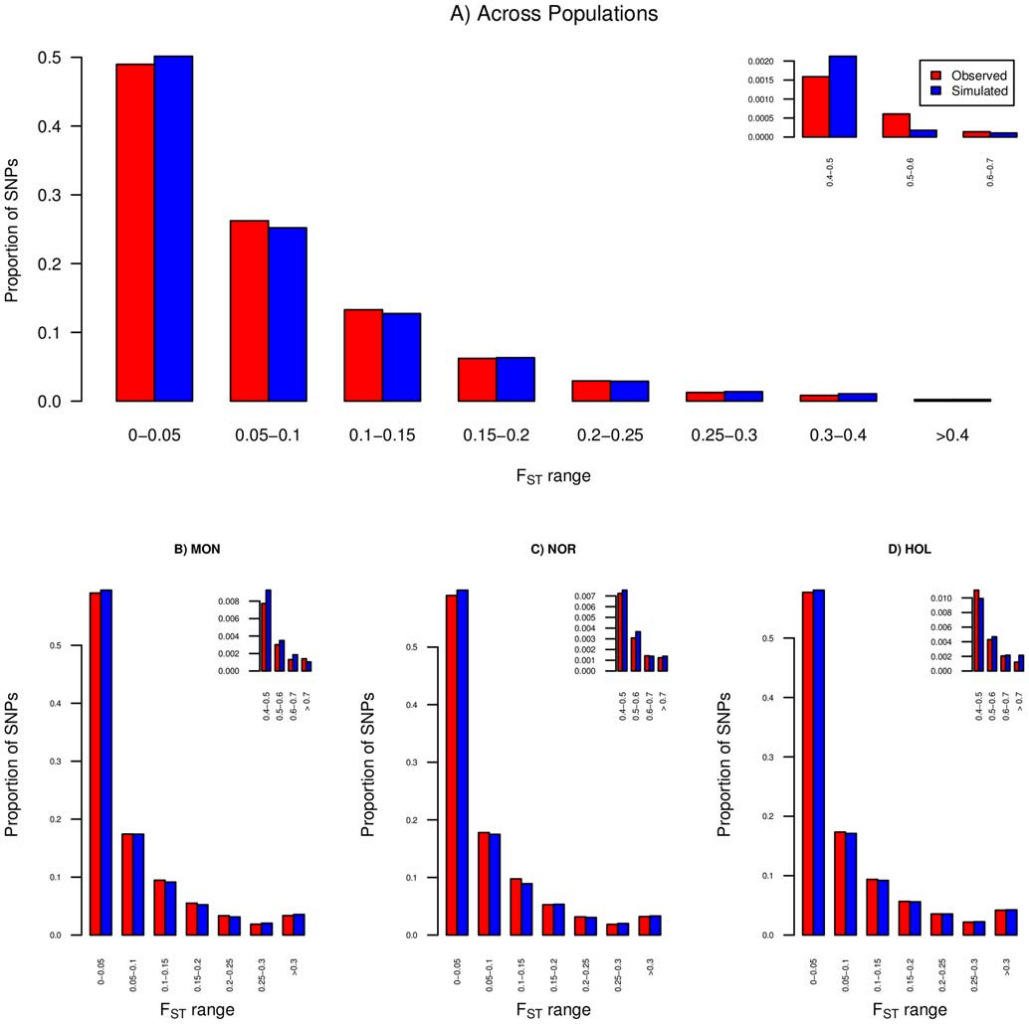
Observed and simulated distribution of SNP *F_ST_* across (A) and within each of the three breeds (B, C, D).

Distributions of SNP-specific *F_ST_* across and within the three breeds are given in Figure 2 for the real and simulated data set. In all cases an overall good adjustment was observed suggesting that most SNPs might behave neutrally and making it difficult to identify outliers SNPs based solely on the empirical distribution as previously proposed [6]. In addition, due to the low level of differentiation (*F_ST_*<0.1), the mode of the empirical (and simulated) distributions was very close to zero, hindering the identification of SNPs under balancing selection (with low *F_ST_*). Yet, very highly differentiated SNPs (*F_ST_* across breeds >0.5) were overrepresented in the real data set (Figure 2A).

### Identification of loci under selection

**Figure 3:**
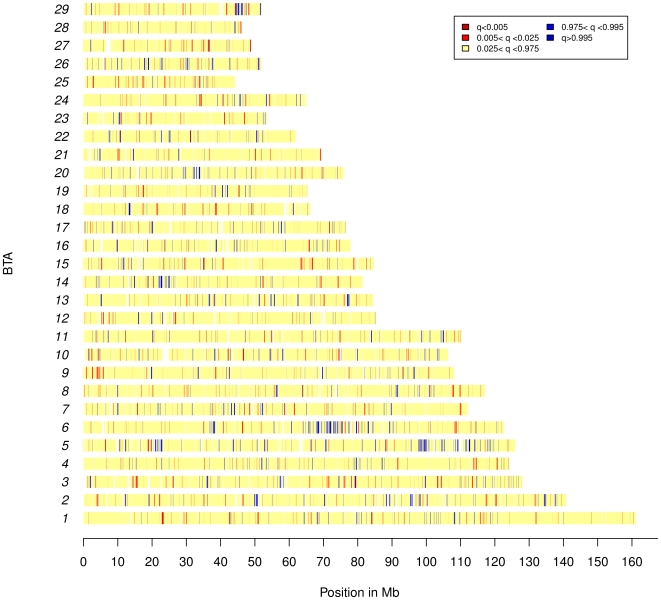
Genome map of differentiated loci. For each SNP the across breed *F_ST_* quantile estimated on the empirical distribution (Figure 2A) is reported according to its chromosomal position.

Selection of a favorable variant is expected to result in a higher level of differentiation for neighboring SNPs. As shown in Figure 3 and detailed in Figure S1, in several instances outlier SNPs tended to cluster to similar regions (e.g. BTA05 or BTA06). At the genome level, nonetheless, the correlation of *F_ST_* between pairs of SNPs as a function of marker distances, both within and across populations, tended to drop quickly toward 0 when SNPs were more than 200 kb apart (Figure S2). This trend of decline was only slightly less pronounced than the one reported for the simulated data set (Figure S2) and similar to the extent of LD within the different breeds [7] in agreement with the hypothesis that most regions might behave neutrally. Hence, in order to identify footprints of selection at the regional level we adopted the strategy proposed by Weir *et al.* [7] consisting in performing average of SNP *F_ST_* over sliding windows. However, because it remains difficult to define, a priori, an optimal window size since it would depend on the strength and timing of selection which are expected to be highly variable, we proposed to smooth SNP-specific *F_ST_* values over each chromosome with a local variable bandwidth kernel estimator (Figures 3, S3, S4 and S5). We also performed this same analysis on the simulated data sets to evaluate to which extent extreme scores are expected under neutrality, allowing in turn the derivation of local q-values. As summarized in Table 2, 13 regions with extreme scores (q-value<0.05) were identified when considering *F_ST_* across populations (Figure 3), 6 of which being also significant within at least one breed (Figures S3, S4 and S5 for MON, NOR and HOL respectively). No additional regions were identified when considering *F_ST_* within each breed which suggested less power to detect footprints of selection using population-specific *F_ST_* estimates.

**Table 2:** Description of the regions under selection based on smoothed *F_ST_* across breeds.

#	BTA	Start-End (peak position) in Mb	*F_ST_* at the peak position (qvalue)	candidate gene	Breeds within which region is also significant
1	3	57.084–58.505 (58.343)	0.375 (0.0298)	CCCBL2	
2	4	78.833–80.43 (79.701)	0.667 (0.0298)	NUDCD3	
3	5	20.301–23.091 (21.02)	0.483 (0.0298)	na	NOR, HOL
4	5	97.803–100.826 (98.26)	0.557 (0.0298)	PIK3C2G	NOR, HOL
5	5	108.461–109.236 (109.182)	0.403 (0.0401)	CD163	
6	5	110.286–111.861 (111.552)	0.46 (0.0435)	ANO2	
7	6	37.433–38.756 (37.963)	0.566 (0.0298)	LAP3/LCORL	MON
8	6	66.599–66.935 (66.809)	0.165 (0.0435)	na	
9	6	68.938–76.32 (72.024)	0.616 (0)	PDGFRA	NOR
10	14	22.02–25.567 (22.634)	0.591 (0)	na	MON, NOR
11	18	12.987–14.058 (13.36)	0.632 (0)	MC1R	MON, HOL
12	20	31.964–33.757 (32.277)	0.523 (0.0298)	GHR	
13	26	22.137–23.191 (22.983)	0.509 (0.0298)	C10ORF76	

**Figure 4:**
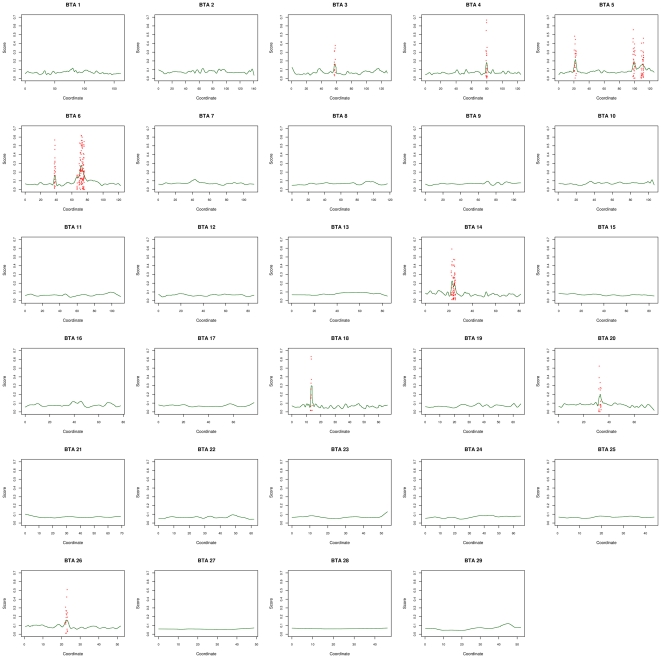
Whole genome map of regions under selection based on the *F_ST_* across populations. For each of the 29 bovine autosomes, the smoothed *F_ST_* is plotted against the chromosomal position (green line). For significant positions (q-value<0.05), non smoothed SNP *F_ST_* are indicated by a red star.

For most of the 13 regions identified, we were able to propose candidate genes on the basis of the gene content in the vicinity of the peak location (Table 2). Interestingly, three of these regions contained or were very close to genes in which mutations have already been related to important function in dairy cattle. For instance, the gene ABCG2 (37.35–37.42 Mb on BTA06) underlying a QTL affecting milk production in Norwegian Red Cattle [18] and in Israeli Holstein [19] was localized about 500 kb upstream the peak of region #7. Nevertheless, the only SNP mapped within this gene could not be considered as an outlier both when considering distribution of *F_ST_* across and within breeds. More recently, a QTL underlying calving difficulty in Norwegian Red cattle was also finely mapped within this same region [20]. In our study, the peak of region #7 was in fact localized within LAP3 (37.96–3798 on BTA06) which was considered as the most likely candidate in this latter study. However, as shown below, LCORL which is localized 200 kb downstream displayed higher evidence of selection, in particular in MON and NOR, and also appear to us as a better candidate since it might control pelvis morphology. Indeed LCORL variants have recently been shown to be strongly associated with hip axis length variation in human [21]. The end of the region #12 was localized 150 kb upstream of GHR (33.89–34.20 on BTA20) (Figure 4D) within which a mutation affecting several milk production traits in HOL [22] and Finnish Ayrshire [23] breeds has been reported. Although not significant when considering *F_ST_* across breeds, 3 SNPs localized within this gene were each displaying high *F_ST_* in one breed (one SNP being in the NOR 1% upper tail distribution). Finally, the interval #11 on BTA18 contained MC1R (13.776–13.778 on BTA18) which is localized within a ∼500 kb gap between two consecutive SNPs (the peak corresponding to the beginning of the gap). MC1R represents an obvious candidate since it determines the ratio of eumelanin and pheomelanin and corresponds to the locus *Extension* involved in coat color in cattle. As reported by Seo *et al.* [24], three alleles have been identified to date in cattle: the *E^D^*, *E^+^* and *e*. The wild-type allele *E^+^* is responsible for combination of red and reddish brown color. Individuals carrying the dominant *E^D^* are black and the recessive *e* allele results in a red color. It has previously been shown that *E^+^*, *E^D^* and *e* are respectively fixed in NOR, HOL and MON [25], in perfect agreement with the absence of significant signal of differentiation in NOR while it was significant in HOL and MON (Table 2). Because of the primary importance of the coloration pattern for herd-book registration, underlying genes might have been among the first to be under (very strong) selection immediately after the definition of breed standard. This latter result might thus be viewed as a proof of concept validating the approach.

### Identification and functional analysis of genes under selection

For most of the regions identified above, it remained difficult to propose candidate genes explaining the observed pattern of region differentiation. Moreover, focusing on large regions might only capture the strongest and/or most recent selection events. As previously mentioned, unexpectedly high (low) level of SNP differentiation might be interpreted as positive (balancing) selection of the underlying genes. We thus computed scores for each annotated RefSeq (see Methods) based on the *F_ST_* of their representative SNP(s) both across and within breeds. In addition, significance of the departure of these scores from the value expected under the neutral hypothesis was further evaluated by considering simulated distributions. For each RefSeq, p-values of the different scores adjusted for multiple testing [26] are reported in Table S2. When controlling the global FDR at the 20% threshold within each breed, we identified 91 RefSeq significant in at least one breed (86 under positive and 6 under balancing selection). More precisely, 20 (0), 25 (4) and 31 (2) RefSeq were detected under positive (balancing) selection in HOL, MON and NOR respectively, nine being shared by NOR and MON. When considering the score based on the *F_ST_*, across breeds only one additional RefSeq score was found significant while more than half of the 91 previously identified ones were also significant. The 43 annotated genes underlying these different RefSeq are given in Table 3. Most of them were physically distantly related and only 14 (33%) were located within the 13 regions identified above (Table 2). Out of the seven regions represented, three were represented by only one gene (NUDCD3 for region #2, WDR51B for region #3 and CD163 for region #5).

**Table 3:** Genes underlying RefSeq found under positive or balancing selection (corrected p-values<0.2) across breeds (indicated by *) or within HOL, MON or NOR (indicated by the corresponding breed name).

Gene (number of underlying RefSeq)	Position in Mb	Region (Table 2)	Significant score (positive selection)	Significant score (balancing selection)
MED12L (1)	BTA1:118.233–118.642		MON	
CNTNAP5 (3)	BTA2:79.729–80.761		HOL*	
UBR4 (1)	BTA2:138.077–138.214		HOL	
FAM40A (2)	BTA3:36.051–36.07		NOR*	
C1ORF123 (2)	BTA3:99.723–99.741		MON	
AGBL4 (2)	BTA3:103.227–104.637		NOR	
NUDCD3 (2)	BTA4:79.666–79.736	2	HOL*	
SYT1 (1)	BTA5:9.355–10.991		NOR	
PPP1R12A (2)	BTA5:11.278–11.447			NOR
WDR51B (2)	BTA5:21.893–22.053	3	NOR*	
MUC19 (1)	BTA5:43.716–43.899		NOR*	
PLCZ1 (2)	BTA5:98.02–98.073	4	NOR	
PIK3C2G (3)	BTA5:98.088–98.641	4	NOR*	
CD163 (4)	BTA5:109.175–109.211	5	NOR	
CCND2 (2)	BTA5:112.625–112.653		MON*	
LAP3 (2)	BTA6:37.962–37.987	7	MON*	
LCORL (3)	BTA6:38.199–38.378	7	MON/NOR*	
KCTD8 (2)	BTA6:65.617–65.881		MON/NOR*	
FRYL (2)	BTA6:69.759–70.029	9	NOR*	
SCFD2 (3)	BTA6:71.168–71.566	9	MON/NOR*	
PDGFRA (2)	BTA6:72.299–72.346	9	MON*	
KIAA1211 (1)	BTA6:74.151–74.298	9	*	
SRD5A2L2 (2)	BTA6:83.061–83.252		NOR*	
FER (4)	BTA7:109.803–110.277			MON*
EPB42 (3)	BTA10:38.36–38.38		NOR	
TSHR (2)	BTA10:95.115–95.25		HOL	
EML5 (2)	BTA10:103.345–103.505		MON*	
KIAA1217 (1)	BTA13:24.029–24.91		NOR	
NCOA3 (3)	BTA13:76.95–77.072		NOR	
KIAA0146 (1)	BTA14:18.787–19.064		HOL	
XKR4 (2)	BTA14:22.691–22.808	10	MON*	
FAM110B (2)	BTA14:24.095–24.237	10	MON	
TOX (2)	BTA14:24.763–25.075	10	MON/NOR*	
KIF1B (4)	BTA16:40.119–40.27		HOL	
PRDM16 (3)	BTA16:46.921–47.267		MON	
RPS6KC1 (1)	BTA16:68.227–68.431		HOL	
ELF2 (2)	BTA17:19.903–19.976		MON*	
ZNF605 (1)	BTA17:46.454–46.486		MON	
GABRG3 (1)	BTA21:3.054–3.828		NOR	
DLGAP1 (6)	BTA24:38.968–39.268		HOL*	
CSMD1 (1)	BTA27:2.108–2.51		MON	
ROBO3 (1)	BTA29:29.794–29.886		NOR	
CAPN1 (3)	BTA29:45.215–45.242		MON*	

Further details are provided in Table S2.

**Figure 5:**
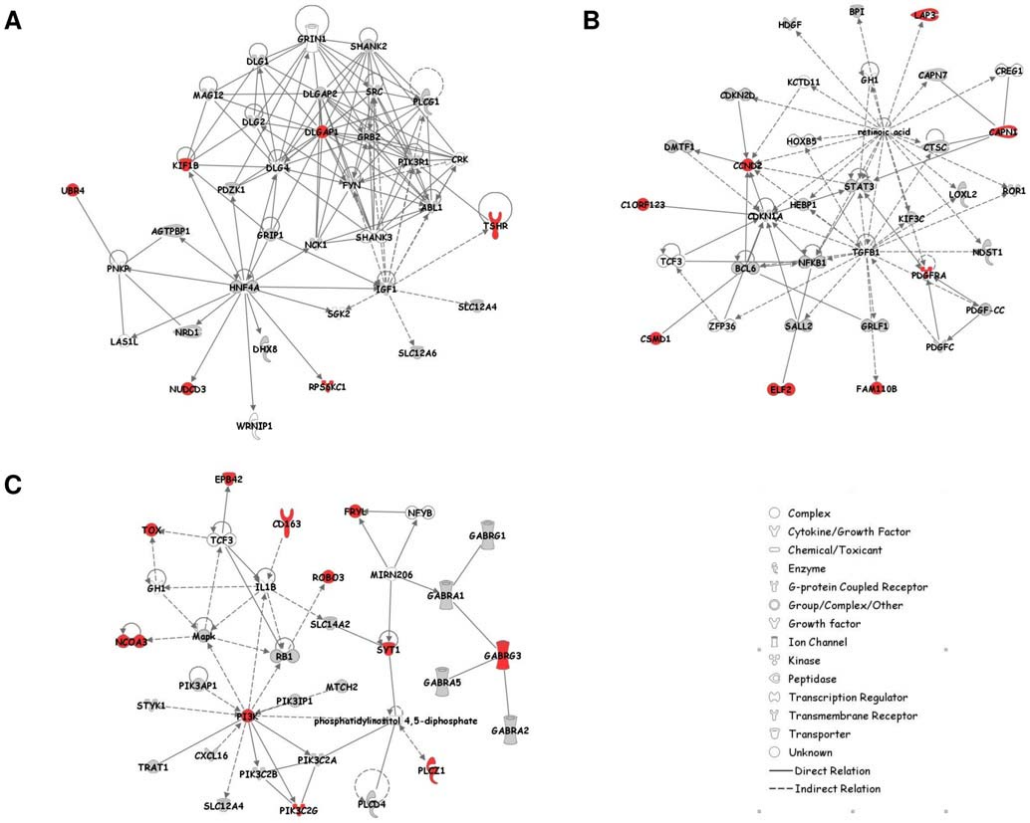
Representation of the gene networks N_MON (A), N_NOR (B) and N_HOL (C). Symbols corresponding to candidate genes are colored in red. Genes colored in grey were represented in our study but did not display any evidence of selection.

**Figure 6:**
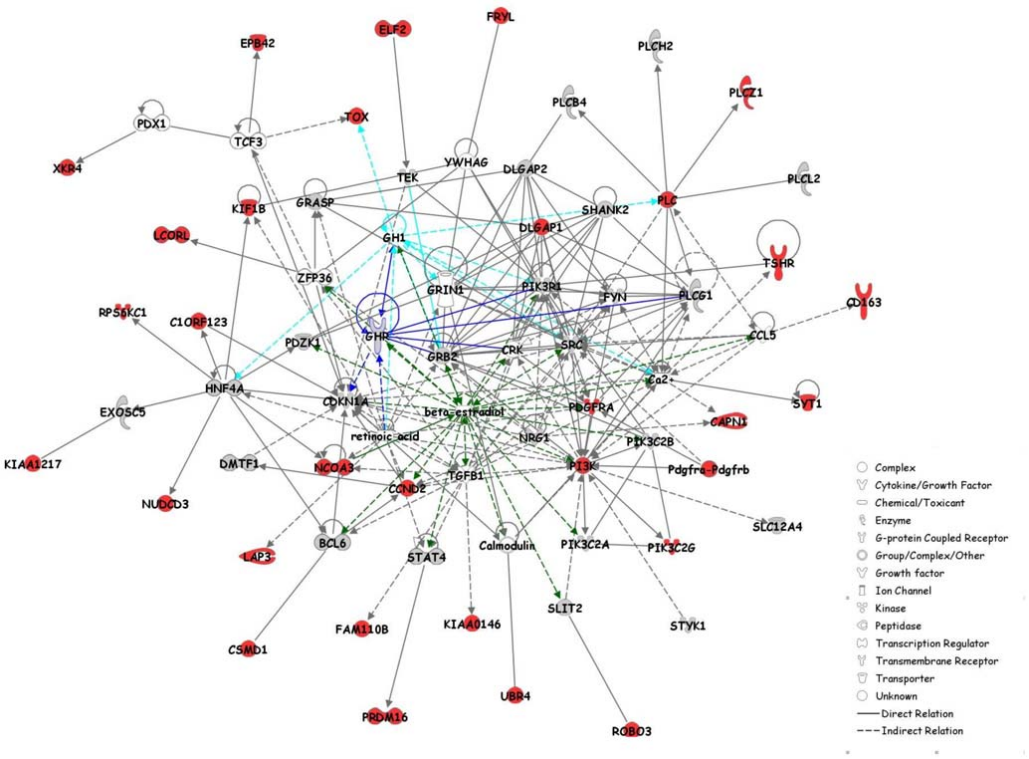
Representation of the gene network GN. Symbols corresponding to genes under selection are colored in red. Genes colored in grey were represented in our study but did not display any evidence of selection. Links between GH1, GHR and β-estradiol and other GN molecules are colored in light blue, blue and green respectively.

In order to characterize the main physiological pathways underlying genes harboring footprints of positive selection we carried out a network analysis under a systems biology framework (see Methods). Indeed, because HOL, MON and NOR have been selected according to a similar breeding goal, we speculated that genes identified could be involved in few biological networks. We first performed three separate network analyses for each breed-specific gene sets. For HOL, MON and NOR respectively 7 (out of 8), 13 (out of 17) and 14 (out of 19) genes were eligible for network analysis leading to the identification of only one significant network per breed (N_HOL, N_MON and N_NOR respectively) (Figure 5). N_HOL was centered on HNF4, DLGAP1 and IGF1, N_MON on TGFB1, retinoic acid and CDKN1A and N_NOR on PI3K and IL1B. Interestingly, although no genes under positive selection were in common between these three different networks, both N_MON and N_NOR contained the Growth Hormone gene (GH) while N_HOL contained the Insulin Growth Factor gene (IGF1). IGF1 and GH represent key molecules of the somatotropic axis which controls milk production, lipolysis and tissue maintenance [27]. In particular, in the mammary gland, GH induces an increase of blood flow and synthesis and a decrease of involution. Hence these results suggested that similar biological pathways were targeted within the three breeds. This led us to extend these network analyses by considering jointly all the 40 genes displaying footprints of positive selection in at least one breed. Two highly significant and interconnected networks were then identified and further merged into a single global network termed GN in the following (Figure 6). Only three genes (WRD51B, KCTD8 and GABRG2) among the 31 eligible ones were not included in GN.

As expected, GN contained several genes involved in the somatotropic axis. In addition to GH1, other important molecules participating to the different GH signal transduction pathways [28] belonged to GN such as GRB2, PLCH2, PLCL2, PLCB4, PLCG1 and SRC or PIK3C2G and PLCZ1, these two latter displaying footprints of selection. GN also contained TGFB1 which is an intramammary auto/paracrine inhibitor of mammary epithelial cells growth and an inducer of apoptosis, which plays a critical role during mammary gland involution [29]. Note that in bovine mammary epithelial cells, GH is also able through its interaction with GHR to suppress expression of TGFB1. Although GHR appeared as a good candidate to explain the signal observed for the region #12 (see above) it was not included in our network analysis but can be connected to GN through 6 molecules (Figure 6).

Besides, several GN molecules are involved in the gonadotropic axis, in particular through the β-estradiol which is a key driver of reproduction. Hence β-estradiol could be connected to 21 GN molecules (Figure 6), four of them (CCND2, NCOA3, PDGFRA and PIK3C2G) being specified by genes displaying footprints of positive selection. Among these four latter genes, NCOA3 interacts with estrogen receptors in a ligand-dependent fashion, enhanced estrogen-dependent transcription and may contribute to development of steroid-dependent cancers [30]. PIK3C2G (such as SRC and STAT4) belongs to PI3K/AKT signaling pathway, one of the main signaling cascades activated by the non genomic activity of estrogen/estrogen receptor [31]. In the bovine mammary parenchyma, in particular, PI3K/AKT was recently demonstrated to be regulated by estrogen [32]. Similarly, this previous study also identified two estrogen regulated networks centered on CDKN1A and TGFB1 respectively, which are both present in GN but did not display signal of positive selection. In addition to their connection with β-estradiol, CCND2 and PDGFRA are also associated with cell proliferation or cell death. In particular, an effect of CCND2 on mammary gland development during pregnancy and involution was demonstrated in transgenic mice [33]. Note that among the three genes under positive selection not included in GN, GABRG2 is a receptor of gamma-aminobutyric acid, one of the mediators of β-estradiol action in brain [34]. Finally, both GH and β-estradiol can regulate level of calcium, a key molecule involved in milk metabolism. Interestingly, GN contained several other molecules related to calcium metabolism such as CAPN1, SYT1 and PLCZ1 which are specified by genes under positive selection. More precisely, CAPN1 is an intracellular protease that requires calcium for its catalytic activity and SYT1 is a calcium sensor in neurotransmitter release [35]. PLCZ1 participates to the PLC/PKC signaling pathway [28] used by both estrogen/estrogen receptors and GH/GHR.

Some of the genes under positive selection belonging to GN could also be related to functions not directly associated with metabolism or reproduction. In particular, CD163 localized under the peak of the previously identified region #5 (Table 2), is involved in innate immune response and clearance of plasma hemoglobin [36]. Similarly, XKR4, TOX and EPB42 are related to erythrocyte structure and functions. Indeed, XKR4 belongs to the Kell blood group complex, TOX variants are associated with HbF levels in sickle cell anemia [37] and EPB42 variants with erythrocytes membrane abnormalities such as hereditary spherocytosis [38]. Notice that some other blood group antigens have been shown to be subjected to balancing selection in human populations [39], [40]. In our study, two genes were found under balancing selection (PPP1R12A in NOR and FER in MON) but not included in the network analysis. Among these, only PP1R12A, a protein phosphatase, can be connected to GN via three molecules (YWHAG, GRB2, FYN) while FER is a tyrosine kinase with a putative role in the regulation of innate immune response [41].

Overall, most of the genes under selection were found to be involved in the gonadotropic system, a key driver of reproduction, and somatotropic system which affects in particular milk metabolism. The antagonistic relationship between milk production and reproductive performances has been largely reported in highly producing dairy cows [4], [5], [42], [43]. In the three breeds considered, artificial selection which might have targeted most of these genes was mainly oriented towards improvement of milk production. Our results thus illustrate how both milk metabolism and reproduction physiological pathways are inter-related at the genetic level. Such relationships might represent one of the main constitutive barriers preventing efficient selection on both traits. In addition, although centered on the same physiological pathways, set of differentiated genes were almost not overlapping among the breeds. This suggests a kind of plasticity in the genome allowing different solutions to respond to a similar breeding goal.

## Methods

### Ethics Statement

DNA needed for the study was previously extracted from commercial AI bull semen straws. No ethics statement is thus required.

### Genotyping data and quality control

A total of 2,803 AI bulls (1,578 from HOL, 641 from NOR and 584 from MON) were genotyped on the Illumina BovineSNP50 chip assay [44] at the Centre National de Génotypage (CNG) platform (Evry, France) using standard procedures (http://www.illumina.com). As detailed in Table S1, these bulls were organized within each breed into large half-sib families of identical sire, a pedigree structure common in dairy cattle because of the widespread use of AI [45]. Bulls were born within a period covering less than 20 years, corresponding to about two generations. Pedigree information was available for more than 6 generations for most individuals allowing the computation of inbreeding coefficient using standard approaches [46]. The within-breed average inbreeding coefficient among the different bulls (<0.05 in the three breeds) was in agreement with those previously reported in the corresponding whole populations [47]. Thirteen animals genotyped on less than 90% of the SNPs were discarded from further analysis. Among the remaining individuals, 26 pairs appeared redundant (>99.9% of identical SNP genotypes). Ten of these pairs clearly corresponded to actual twins (recorded as full sibs and probably resulting from embryo transfer manipulations) allowing assessment of the genotyping error rate at 0.06%. Only one individual per pair was kept for further analysis. For the 16 other pairs, individuals were declared as half-sibs and might correspond to sample duplication (their DNA was extracted approximately at the same time). The 32 corresponding individuals were thus discarded.

A total of 728 SNPs mapping to the X chromosome and 1,672 other SNPs (∼3%) which were genotyped on less than 90% of the individuals in at least one breed were not considered in the analysis. An exact test for Hardy-Weinberg Equilibrium (HWE) [48] was carried out within each breed separately on the 51,601 remaining SNPs. Based on the obtained p-values, q-values [49] were estimated for each SNP using the R package qvalue (http://cran.r-project.org/web/packages/qvalue/index.html). A total of 829 SNPs exhibiting q-value<0.01 in at least one breed were then discarded from further analysis.

### Estimation of allele frequencies

Although the number of families and individuals considered within each breed was large, we took into account the half-sib pedigree structure in estimating population allele frequencies by considering only maternally inherited allele. Indeed, dams were more representative of the population and less related than the bulls (Table S1). Within each breed, population allele frequencies were then estimated by a simple counting algorithm run iteratively. At each step, the most likely sire genotypes were first estimated conditionally on the bull genotypes and allele frequencies (estimated for the first step by simple counting on all bulls) and allowing a 1% genotyping error rate. Each maternally inherited allele was then identified to update population allele frequencies. The procedure was stopped when no change in deduced genotypes for all sires was observed. Because genotyping data were available for 14 out of the 64 bull sires considered, we could estimate the prediction error rate as being equal to 1.2% (assuming no genotyping error in genotyping data), similar rates being observed on simulated data. This procedure also allowed computing the number of mendelian inconsistencies which was found similar to the observed genotyping error rate as estimated above (0.02% versus 0.06%). Thus, allele frequencies could be considered as estimated with high precision and relied upon on average 485 (from 281 to 579) maternally inherited alleles in MON, 520 (320–635) in NOR and 1,293 (882–1293) in HOL. Finally, only SNPs displaying a MAF above 0.001 in the three breeds were retained for further analysis resulting in a total of 42,846 SNPs.

### Population Structure

Assessment of population structure was performed by the standard unsupervised Bayesian clustering approach implemented in the software STRUCTURE 2.2 [50]. Among the 42,846 available SNPs, only 8,342 SNPs (selected to achieve a minimal inter-marker distance above 200 kb) were included in the analysis and marker position information was considered. STRUCTURE 2.2 was then run 3 times with a single prior value of K = 3 for the number of clusters, and a burning period of 5000 iterations followed by 10,000 iterations. The three replicated analyses were then aligned using default options of the CLUMPP software [51].

### Estimation of F_ST_

Estimation of SNP and population-specific *F_ST_* were based on the pure drift model proposed by Nicholson *et al.* [15] which allowed relaxing the assumption of an identical level of differentiation across populations [52]. Briefly, the frequency α_ij_ of a given reference allele at SNP *i* within population *j* is modeled as a truncated Gaussian: α_ij_ ∼ *N*(*π_i_* , *c_j_ π_i_* (1-*π_i_* )) where *π_i_* can be interpreted as the frequency of the allele in the population ancestral to the three breeds considered and *c_j_* represents a differentiation parameter (relatively to the ancestral population) analogous to a *F_ST_* coefficient for low level of differentiation [15]. These authors proposed a standard method of moments' estimator for *c_j_* as 
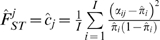
 where 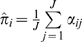, *I* represents the total number of SNPs and *J* the total number of populations. Note that 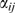 corresponds to the observed allele frequencies and no correction for sample size was performed since sample sizes were large (see above). Based on simulated data, Nicholson *et al.* [15] showed that the method of moments estimator of *c_j_* performed well providing levels of differentiation are similar across populations. We further simply estimated the 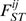 for SNP *i* within population *j* as 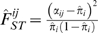 and across populations as 
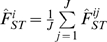
.

### Mapping information and anchorage of SNPs

Among the 42,846 SNPs, 41,777 mapped to a bovine autosome on the latest bovine genome assembly Btau_4.0 (http://www.hgsc.bcm.tmc.edu/projects/bovine/), the others 1,069 belonged to unassigned contigs. As shown in Table S3, on average one SNP every 60.8 kb (from 53.7 kb on BTA25 to 72.6 kb on BTA05) was available allowing dense and homogeneous genome coverage. More precisely, few large gaps remained since only 2.81% of the inter-marker intervals are larger than 200 kb (the size of the larger gap being 2 Mb) and the 99^th^ (95^th^) percentile of the inter-marker distance distribution was equal to 278 kb (159 kb). Conversely, few short gaps between successive SNPs were observed: the 1^st^ (5^th^) percentile of the inter-marker distance distribution being equal to 20.1 kb (21.7 kb).

### Simulated data set

Simulations were carried out using the coalescent program GENOME [53] to obtain the genome-distribution of parameters of interest under a selectively neutral model. For each population 100 individual genomes consisting of 29 100-Mb chromosomes were simulated, each being composed of 4,000 segments separated by 25 kb (assuming a recombination rate of 10^−8^ per bp). To reflect breed formation the demographic scenario consisted in three completely isolated populations separated t = 25 generations ago from an initial common population with an effective population of N_e_ = 5,000. Note that under the pure drift model described previously, the population specific differentiation parameter 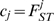 might be thought of as the inverse of an effective bottleneck size *c_j_ = t/N_j_* where *N_j_* represents the (haploid) effective population size of population *j* [15] providing a natural estimate in our demographic scenario for each population size after their splitting. However because our 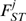 estimator is somewhat downwardly biased, these population sizes needed to be adjusted until matching of the observed and simulated *F_ST_* both computed within and across populations. Effective chromosome size (twice the effective population size) retained for simulations was respectively 215, 220 and 180 for MON, NOR and HOL. Finally, for each chromosome we fixed the number of mutations to 4,000. The resulting 126,000 SNPs were subjected to the same ascertainment scheme as the one adopted for real data (MAF>0.001 in the three populations) leading to a simulated data set containing 55,591 SNPs. Marker coverage was close to the real data set one (on average one SNP every 51.8 kb) with a similar proportion of large gaps (only 3.04% above 200 kb). The estimated *F_ST_* across the three simulated populations was 0.0707 and respectively 0.0693, 0.0683 and 0.0745 for the simulated MON, NOR and HOL populations, almost identical to the one computed on the real data sets (see Results).

### Identification of regions under selection

In order to identify regions under selection (with an unexpectedly high proportion of SNPs subjected to selection), we followed the locally adaptive procedure which allows to account for variations in distance between the different tested positions [54]. Individual SNP *F_ST_* values were first smoothed over each chromosome with a local variable bandwidth kernel estimator [55]. A similar approach was performed on the simulated data sets to estimate the whole genome distribution of the score under the neutral hypothesis. Based on this distribution, local q-values were then calculated using the R package qvalue (http://cran.r-project.org/web/packages/qvalue/index.html) to identify significant outlier regions (q-value<0.05).

### SNP Annotation

Because the annotation of the bovine genome is still sparse, the gene content information was derived from the TransMap cross-species alignments available in the UCSC Genome Browser (http://genome.ucsc.edu/). For closer evolutionary distances, the alignments are created using syntenically filtered BLASTZ alignment chains, resulting in a prediction of the orthologous genes in cow. In total, 46,598 different RefSeq identifiers were anchored in the latest bovine genome assembly (http://genome.ucsc.edu/). Considering that most consecutive SNPs on the map were separated by more than 20 kb and the high correlations between the 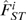 for closely related SNPs (see Results), a SNP was considered as representative of a gene if it was localized within the boundaries positions of the gene extended by 15 kb upstream and downstream. According to this criterion, the 17,833 SNPs out of the 41,777 SNPs were representative of 18,986 different TransMap RefSeq identifiers (out of the 46,598 ones) detailed in Table S2. On average they were represented by 2.30 SNPs (from 1 to 54), 7,723 (41%) being represented by at least 2 SNPs. Subsequent annotation and analyses were carried out with the web-based pathways analysis tool Ingenuity Pathway Analysis (IPA) v7.0 (Ingenuity Systems Inc., USA, http://www.ingenuity.com/). Among the 18,986 different TransMap RefSeq identifiers (see above), 18,944 identifiers (99.8%) were represented in the Ingenuity Pathway Knowledge Base (IPKB) and corresponded to 7,935 different genes further considered as the reference set. Although because of RefSeq redundancy, most SNPs were representative of several RefSeq, only 402 SNPs (out of the 17,806 ones) were representative of more than one gene.

### Identification of genes under selection

A gene could be regarded as being under selection if it contained an unexpectedly high proportion of highly (or lowly) differentiated SNPs. First, each RefSeq was given a score corresponding to the *F_ST_* average of its representative SNPs. If the RefSeq was represented by only one SNP, its score was the corresponding *F_ST_*. To evaluate the distribution under the neutral hypothesis of each score we further draw 50,000 independent samples of 17,806 *F_ST_* (across and within each populations) which were assigned to 18,986 “simulated” RefSeq by exactly mimicking the observed SNP RefSeq content. A p-value was then computed for each RefSeq (both across and within each population) by counting the number of times the observed score was above or below the simulated ones. To deal with multiple testing issues, we further applied a Benjamini and Hochberg correction [26] on the resulting p-values as implemented in the R package qvalue http://cran.r-project.org/web/packages/qvalue/index.html). Note that our strategy considered SNPs are independent from each others which might be reasonable under the null hypothesis of neutrality given our marker density and background LD within the different populations (see Results).

### Networks Analyses of the differentiated SNP

IPA was used to organize genes showing evidence of selection into networks of interacting genes and to identify pathways containing functionally related genes. More precisely, network analysis consists in searching for direct and indirect interactions (known from the literature and manually curated by experts) between candidate genes and all other molecules (genes, gene products or small molecules) contained in IPKB. The complete list of RefSeq identifiers with their respective scores (across and within breeds) were uploaded into IPA and each were mapped to their corresponding IPKB gene object (see above). Candidate genes are eligible for network generation if there is at least one wild type IPKB interacting molecule. Based on the information available for eligible candidate genes (focus genes), IPA further constructs networks by maximizing the number of focus genes and their inter-connectivity in the limit of 35 molecules per network. Note that additional highly connected non focus molecules are also included. Finally, for each network, a right-tailed Fisher exact test is implemented to evaluate how likely the focus genes it contains might be found together by chance. Only those networks with a score (-log(p-value)) greater than 3 were considered as significant. In addition, networks might be inter-connected (sharing at least one molecule) which strengthen the importance for the underlying biological functions. Networks are graphically represented by nodes with various shapes (according to the molecule type) and edges (according to their biological relationships).

## Supporting Information

Table S1 Sample description
(0.00 MB PDF)

Table S2 Description of the results for the 18,986 RefSeq represented in the analysis. For each RefSeq, we report the position on the genome, the underlying gene (based on IPA annotation), the score derived from the FST values of the SNP localized within it and the and p-values corrected for multiple testing for positive and balancing selection tests both across and within each breed. The interval number (Table 2) is reported if the RefSeq is localized within a significant region previously reported.
(1.87 MB ZIP)

Table S3 Genome coverage and SNP density.
(0.01 MB PDF)

Figure S1 Observed FST (across and within the three breeds) for each SNP as a function of chromosome position (one page per chromosome). The red (blue) dashed line corresponds to the 99% (97.5%) threshold on the corresponding empirical distributions.
(1.39 MB ZIP)

Figure S2 Correlation of FST (across and within each of the three breeds) for pairs of markers as a function of physical distances in the real (upper panel) and simulated (lower panel) data sets.
(0.02 MB PDF)

Figure S3 Whole genome map of regions under selection based on the FST within MON. For each of the 29 bovine autosomes, the smoothed FST is plotted against the chromosomal position (green line). For significant positions (q-value<0.05), non smoothed SNP FST are indicated by a red star.
(0.22 MB ZIP)

Figure S4 Whole genome map of regions under selection based on the FST within NOR. For each of the 29 bovine autosomes, the smoothed FST is plotted against the chromosomal position (green line). For significant positions (q-value<0.05), non smoothed SNP FST are indicated by a red star.
(0.22 MB ZIP)

Figure S5 Whole genome map of regions under selection based on the FST within HOL. For each of the 29 bovine autosomes, the smoothed FST is plotted against the chromosomal position (green line). For significant positions (q-value<0.05), non smoothed SNP FST are indicated by a red star.
(0.23 MB ZIP)
