# Supplementary material for: The Genome Response to Artificial Selection: A Case Study in Dairy Cattle
Source: PLoS One. 2009 Aug 12;4(8):e6595. doi: 10.1371/journal.pone.0006595 (PMC2722727; doi:10.1371/journal.pone.0006595)
Supplement: Table S1 — Sample description (0.00 MB PDF) [file pone.0006595.s001.pdf]

Breed	Total Number of AI bulls (Number of Half-sib families)	Average Family Size (Min-Max)	Bull Year of Birth (interval containing 90% of Bulls)	Median Inbreeding among the bulls	Number of different dams	Median Inbreeding among the dams
MON	584 (16)	36.5 (14-88)	1985-2002 (1987-2001)	0.050	504	0.031
NOR	641 (17)	37.7 (11-73)	1987-2002 (1988-2002)	0.046	566	0.035
HOL	1578 (31)	50.9 (21-133)	1981-2003 (1986-2002)	0.049	1355	0.037
