# Supplementary material for: The Genome Response to Artificial Selection: A Case Study in Dairy Cattle
Source: PLoS One. 2009 Aug 12;4(8):e6595. doi: 10.1371/journal.pone.0006595 (PMC2722727; doi:10.1371/journal.pone.0006595)
Supplement: Table S3 — Genome coverage and SNP density. (0.01 MB PDF) [file pone.0006595.s003.pdf]

Bovine Chromosome	Number of SNPs	Chromsome size (in Mb)	Average Marker spacing in kb (standard deviation)	Median Marker spacing in kb (min-max)	Percentage of intervals larger than 200 kb (100 kb)
01	2718	160.91	59.22 (49.64)	41.23 (0.001-632)	2.65 (13.80)
02	2216	140.43	63.40 (62.93)	41.14 (0.001-614)	3.66 (15.08)
03	2094	127.12	60.74 (61.03)	41.55 (0.001-808)	3.06 (13.62)
04	2066	124.01	60.05 (48.66)	42.00 (0.001-362)	2.95 (13.75)
05	1734	125.8	72.59 (76.49)	46.98 (0.147-1120)	6.06 (19.45)
06	2074	122.44	59.06 (58.44)	40.41 (2.659-826)	2.99 (12.20)
07	1816	111.70	61.54 (57.12)	41.23 (2.792-708)	3.09 (14.49)
08	1934	116.93	60.49 (52.80)	42.62 (0.001-738)	2.64 (13.19)
09	1681	108.05	64.31 (61.18)	43.97 (0.449-761)	3.93 (15.54)
10	1772	105.55	59.60 (71.52)	39.60 (0.001-2080)	2.48 (13.55)
11	1839	109.96	59.83 (54.12)	41 (10.175-775)	2.56 (13.60)
12	1337	85.20	63.77 (66.20)	41.81 (0.238-933)	3.22 (15.19)
13	1414	84.08	59.50 (51.64)	42.64 (0.001-701)	2.19 (13.09)
14	1392	81.27	58.43 (48.86)	41.36 (0.21-576)	2.23 (13.59)
15	1360	84.53	62.20 (55.98)	43.53 (0.001-684)	2.72 (14.79)
16	1286	77.8	60.54 (61.80)	41.16 (0.179-1020)	2.72 (13.23)
17	1297	76.35	58.91 (53.99)	41.35 (5.909-840)	1.77 (13.73)
18	1080	66.03	61.20 (60.74)	43.59 (0.772-917)	2.41 (13.35)
19	1122	65.07	58.04 (47.75)	42.56 (1.367-553)	2.05 (11.69)
20	1316	75.41	57.34 (51.44)	40.36 (1.671-837)	1.75 (11.71)
21	1111	69.14	62.29 (58.64)	43.23 (0.001-849)	3.33 (14.41)
22	1044	61.7	59.16 (45.94)	43.82 (0.001-413)	2.01 (13.90)
23	895	53.33	59.65 (53.18)	42.29 (2.21-510)	2.68 (13.53)
24	1053	64.93	61.72 (51.61)	44.07 (12.271-457)	2.47 (14.45)
25	811	43.46	53.65 (40.44)	40.16 (2.005-350)	1.48 (9.51)
26	884	51.26	58.05 (45.53)	43.93 (0.001-683)	1.70 (11.10)
27	802	48.73	60.83 (72.91)	42.97 (4.669-1680)	2.00 (12.73)
28	781	45.98	58.94 (48.35)	42.53 (0.001-411)	2.69 (11.79)
29	848	51.78	61.14 (58.53)	44.27 (0.001-822)	2.60 (12.87)

TOTAL	41777	2539	60.82 (57.31)	42.16 (0.001-2080)	2.81 (13.77)
