# Supplementary material for: The Genome Response to Artificial Selection: A Case Study in Dairy Cattle
Source: PLoS One. 2009 Aug 12;4(8):e6595. doi: 10.1371/journal.pone.0006595 (PMC2722727; doi:10.1371/journal.pone.0006595)
Supplement: Figure S1 — Observed FST (across and within the three breeds) for each SNP as a function of chromosome position (one page per chromosome). The red (blue) dashed line corresponds to the 99% (97.5%) threshold on the corresponding empirical distributions. (1.39 MB ZIP) [file pone.0006595.s004.zip › Supp_FIG1.pdf]

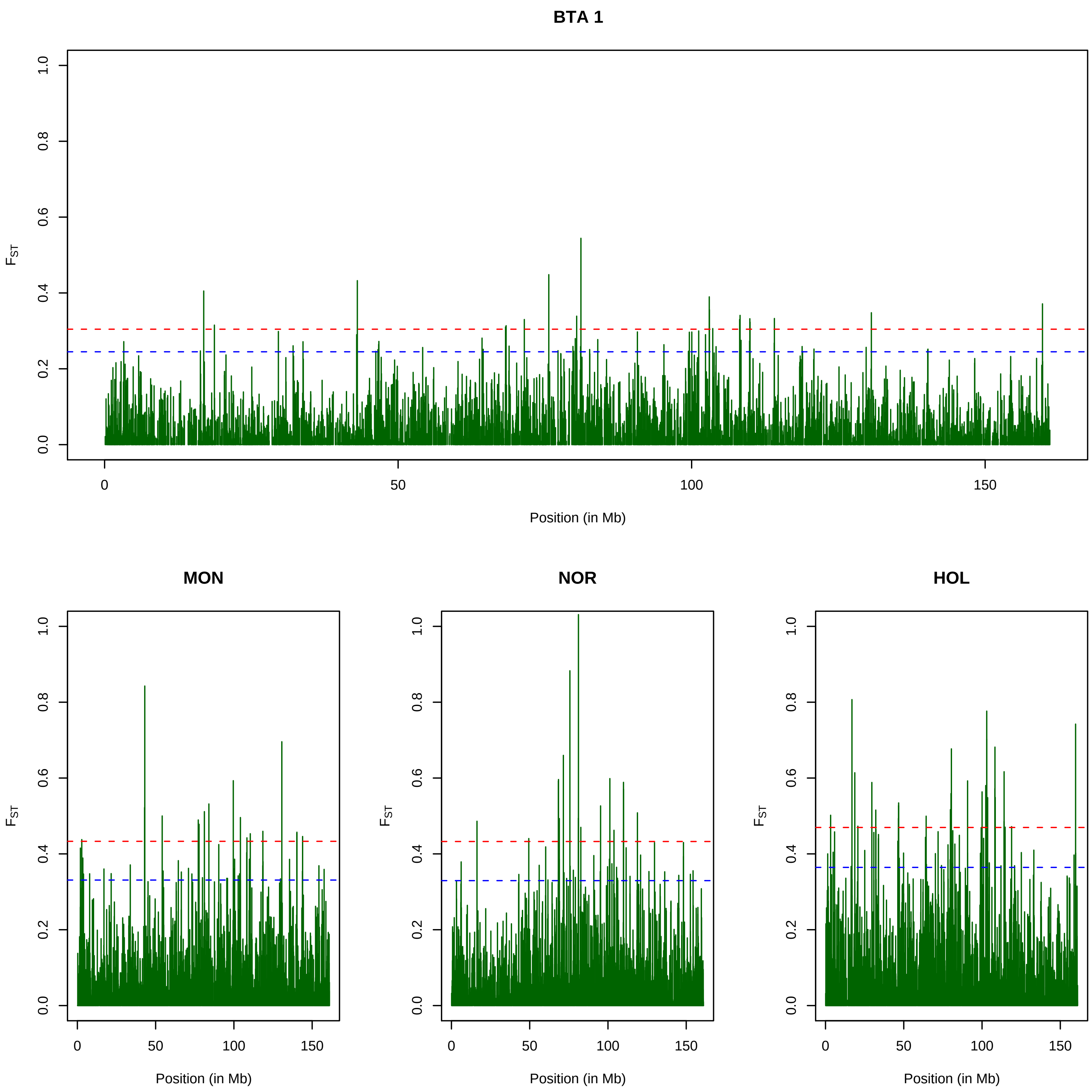

BTA 1
MON
NOR
HOL
F_{ST}
Position (in Mb)
0.0
0.2
0.4
0.6
0.8
1.0
0
50
100
150

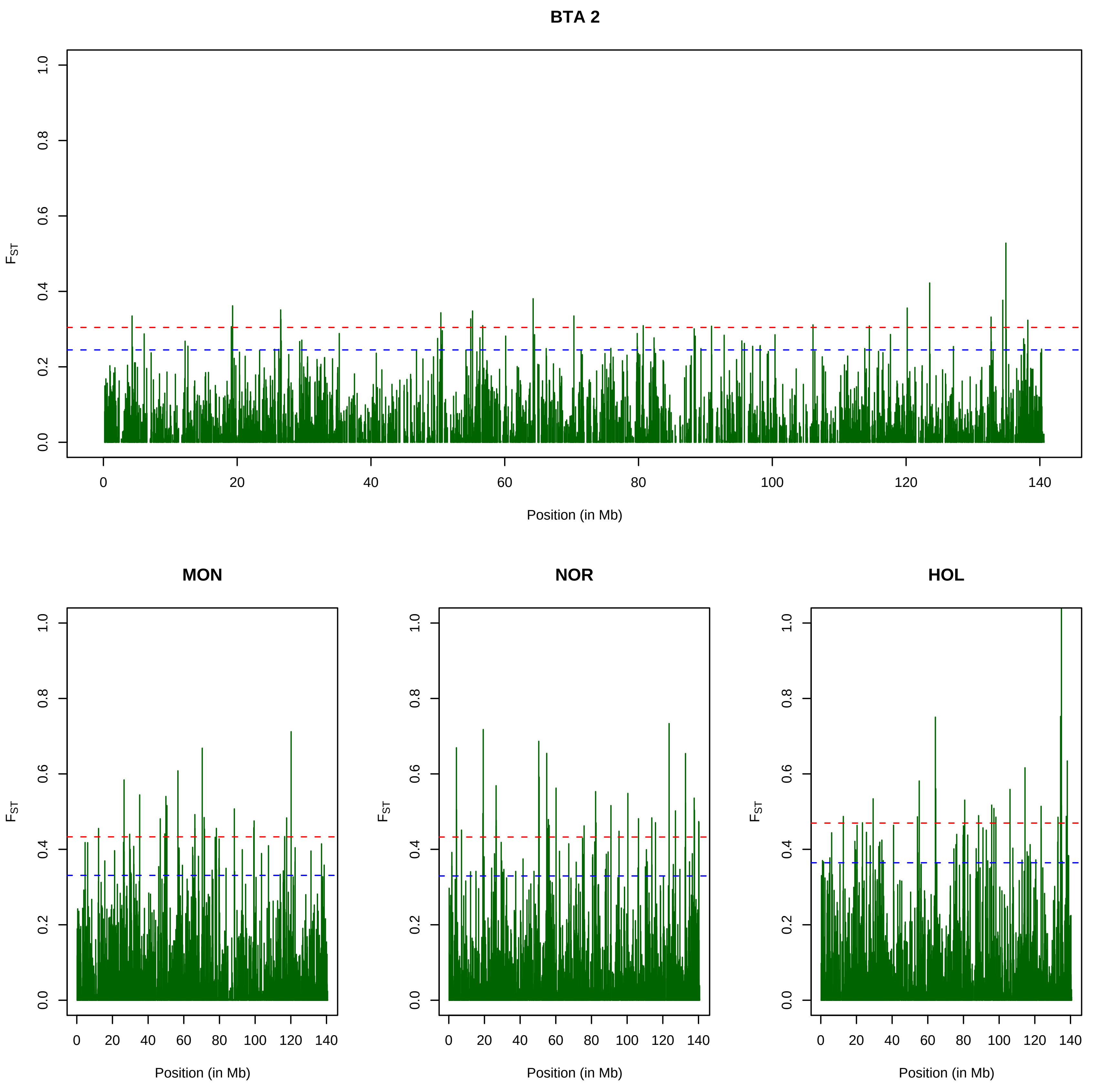

BTA 2
MON
NOR
HOL
F_{ST}
Position (in Mb)
0.0
0.2
0.4
0.6
0.8
1.0
0
20
40
60
80
100
120
140

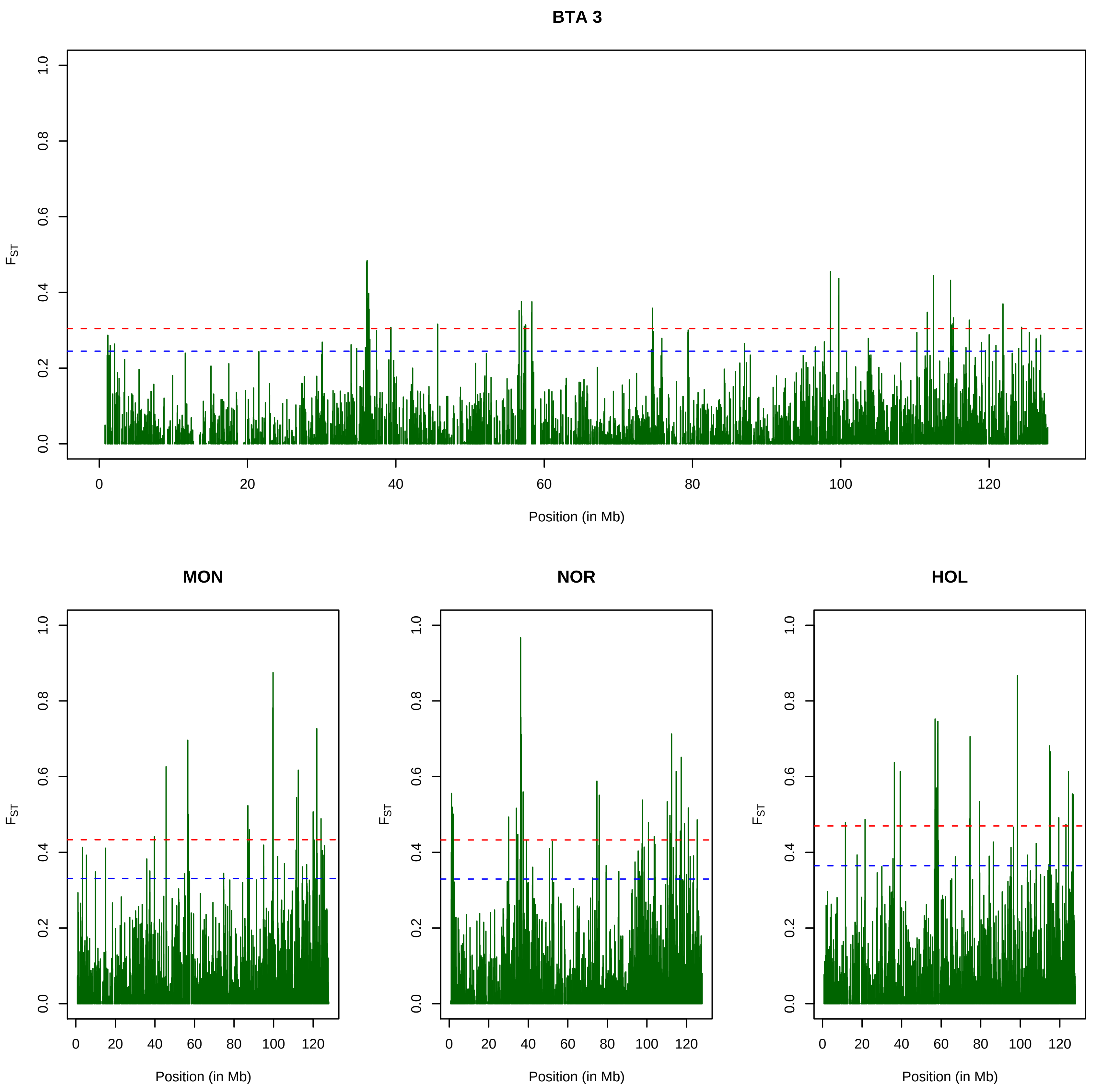
BTA 3
MON
NOR
HOL
F_ST
Position (in Mb)
0.0
0.2
0.4
0.6
0.8
1.0
0
20
40
60
80
100
120

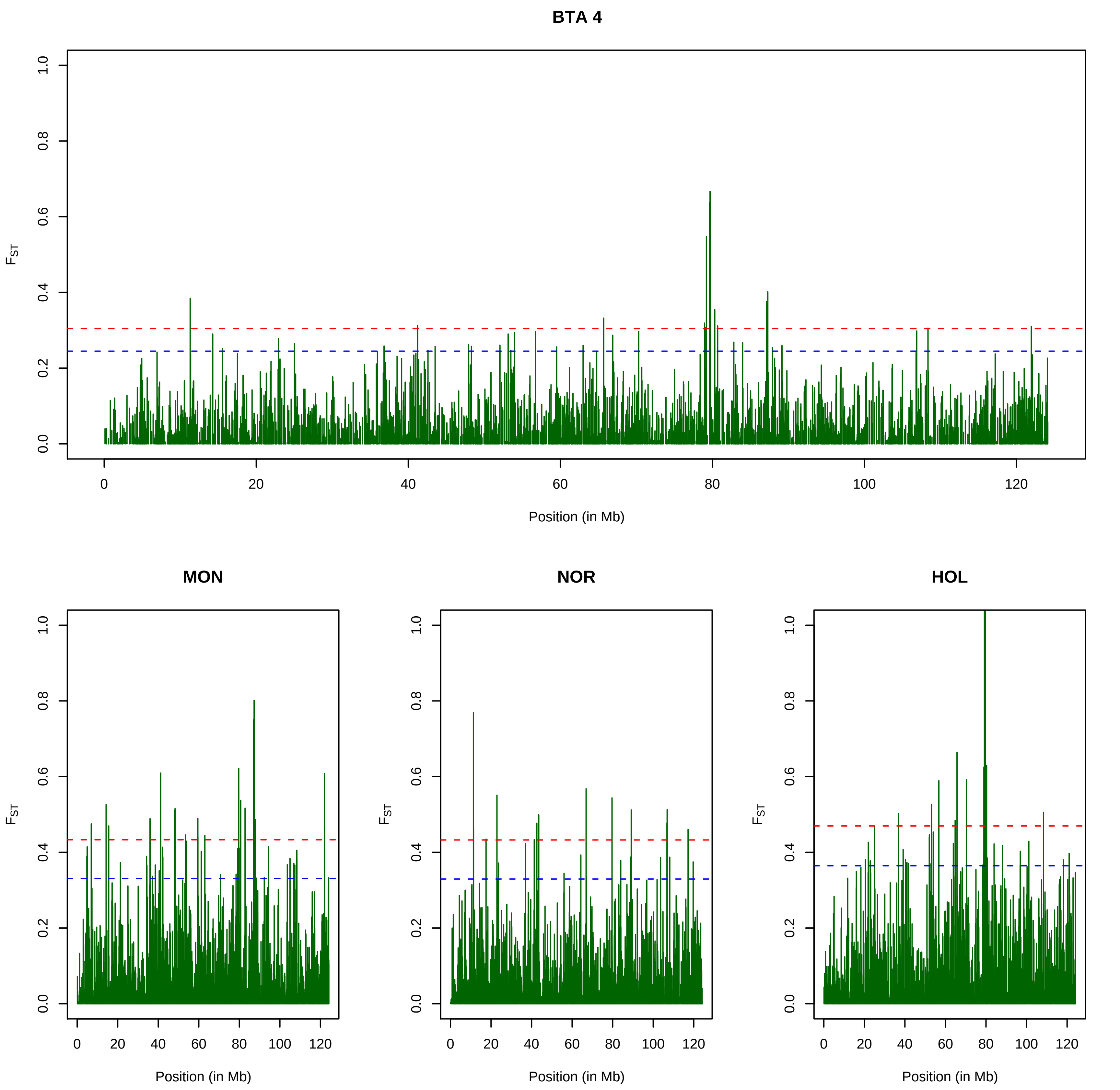
BTA 4
MON
NOR
HOL
F_ST
Position (in Mb)
0.0
0.2
0.4
0.6
0.8
1.0
0
20
40
60
80
100
120

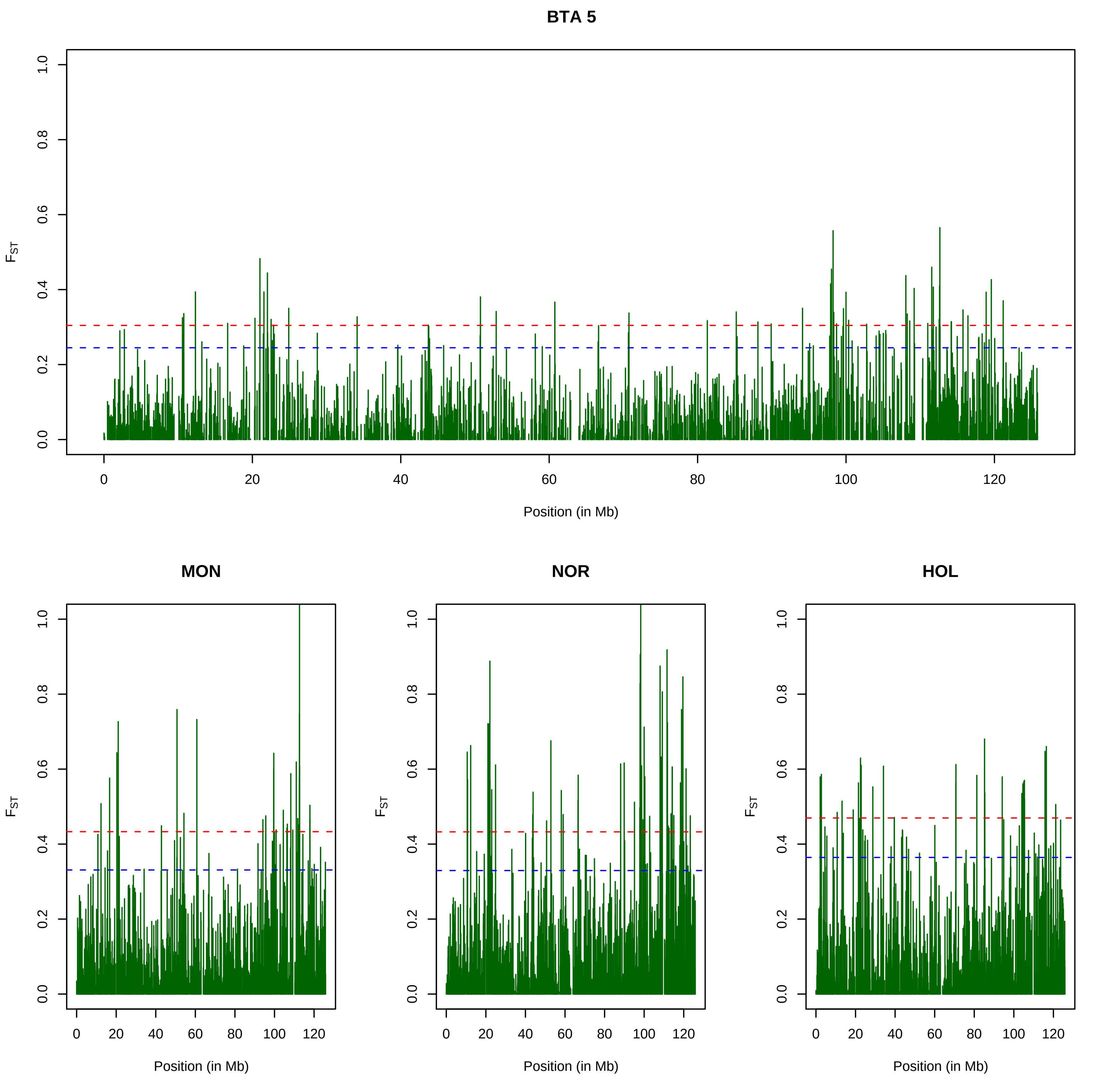

BTA 5
MON
NOR
HOL
F_{ST}
Position (in Mb)

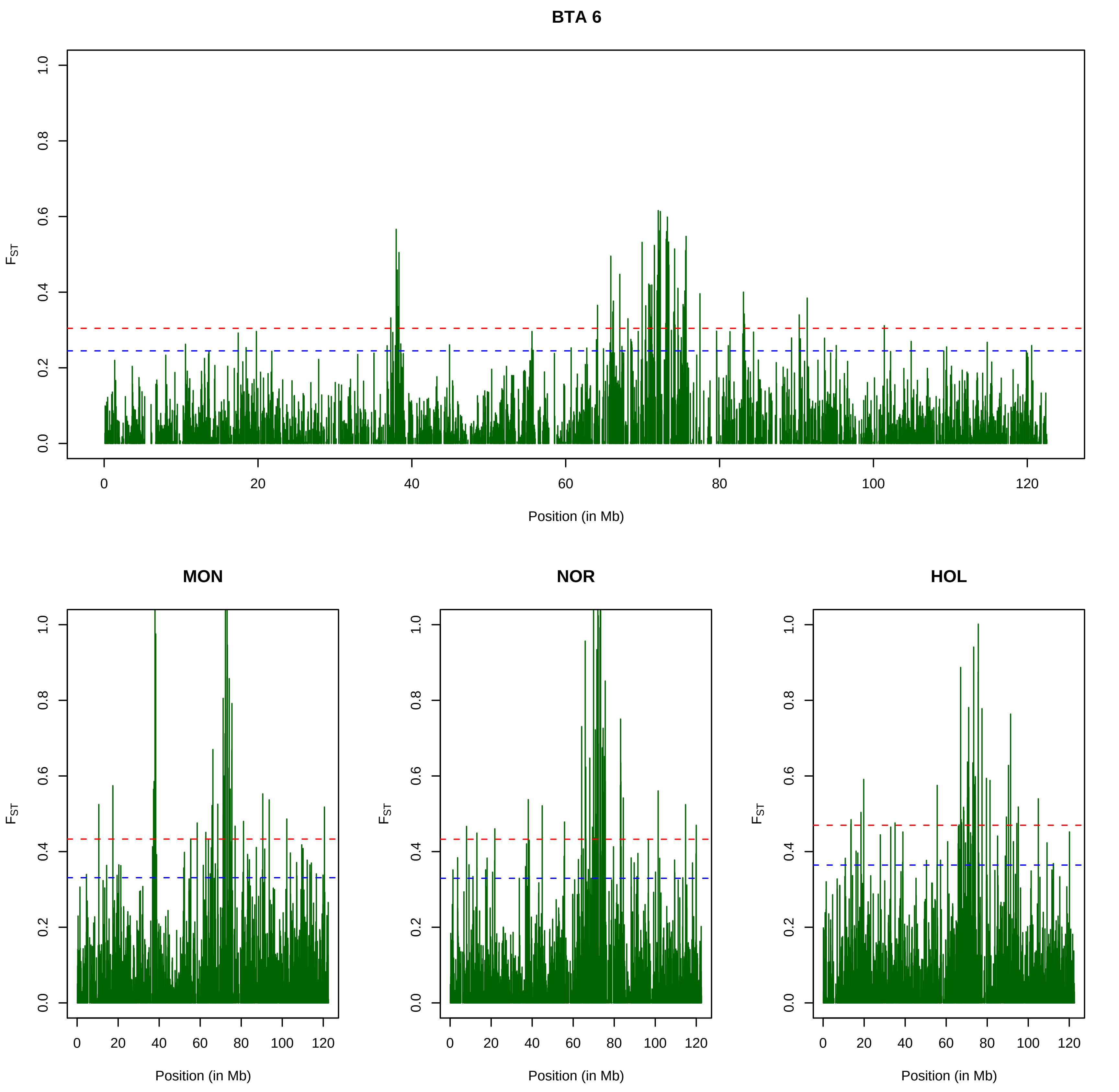
BTA 6
F_{ST}
Position (in Mb)
MON
NOR
HOL
0.0
0.2
0.4
0.6
0.8
1.0
0
20
40
60
80
100
120

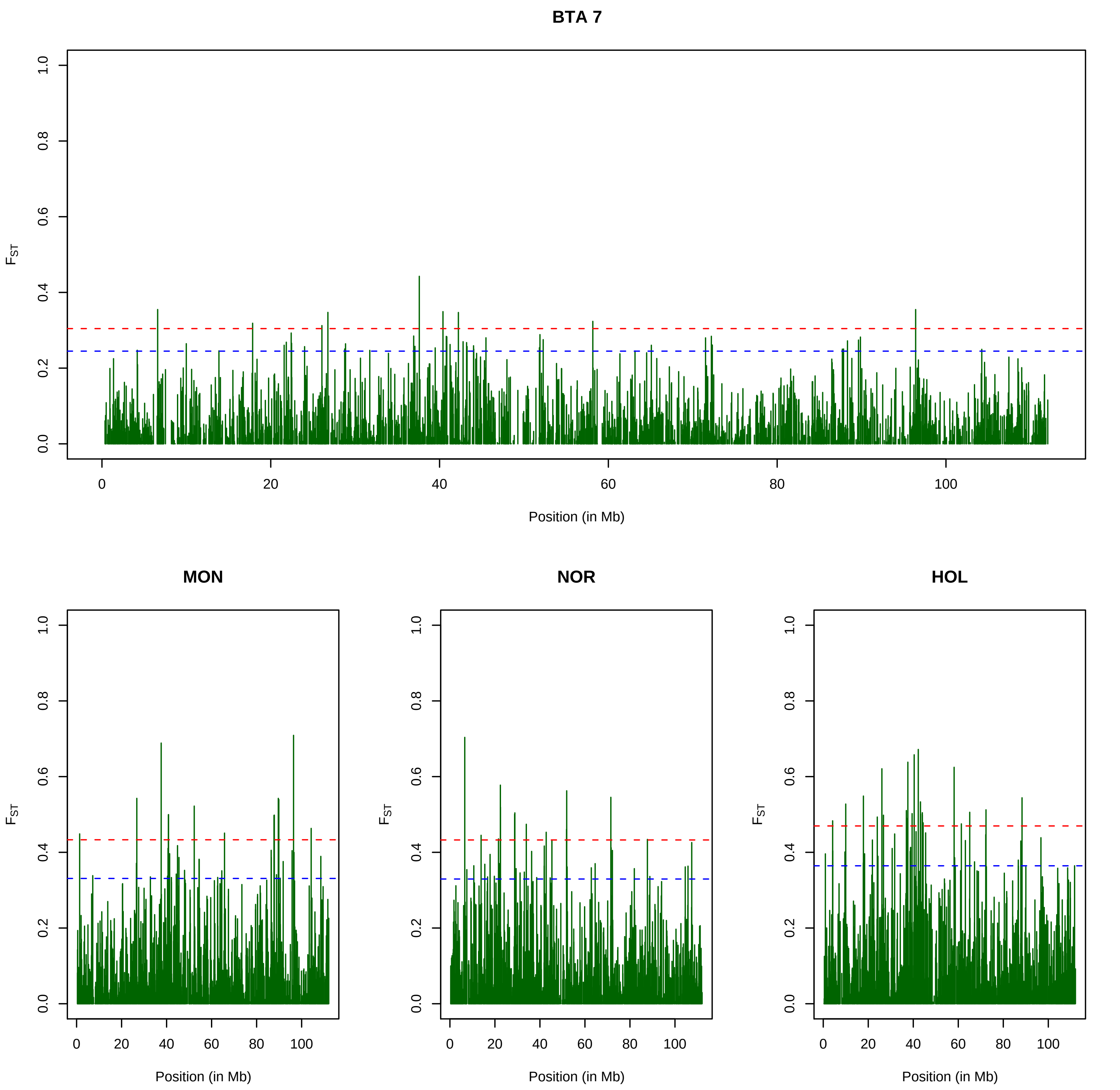
BTA 7
F_ST
Position (in Mb)
MON
NOR
HOL
0.0
0.2
0.4
0.6
0.8
1.0
0
20
40
60
80
100

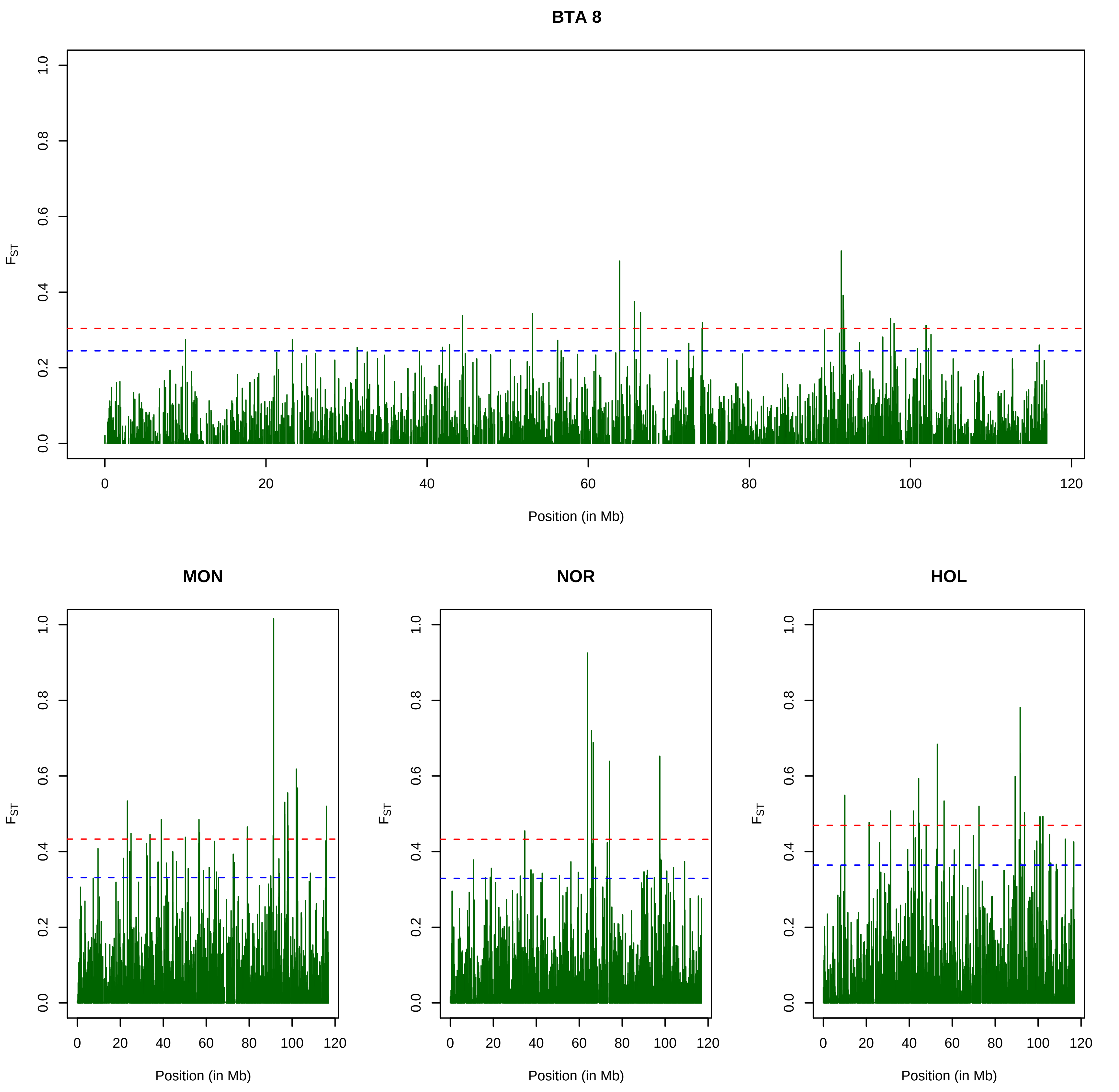

BTA 8
MON
NOR
HOL
F_ST
Position (in Mb)

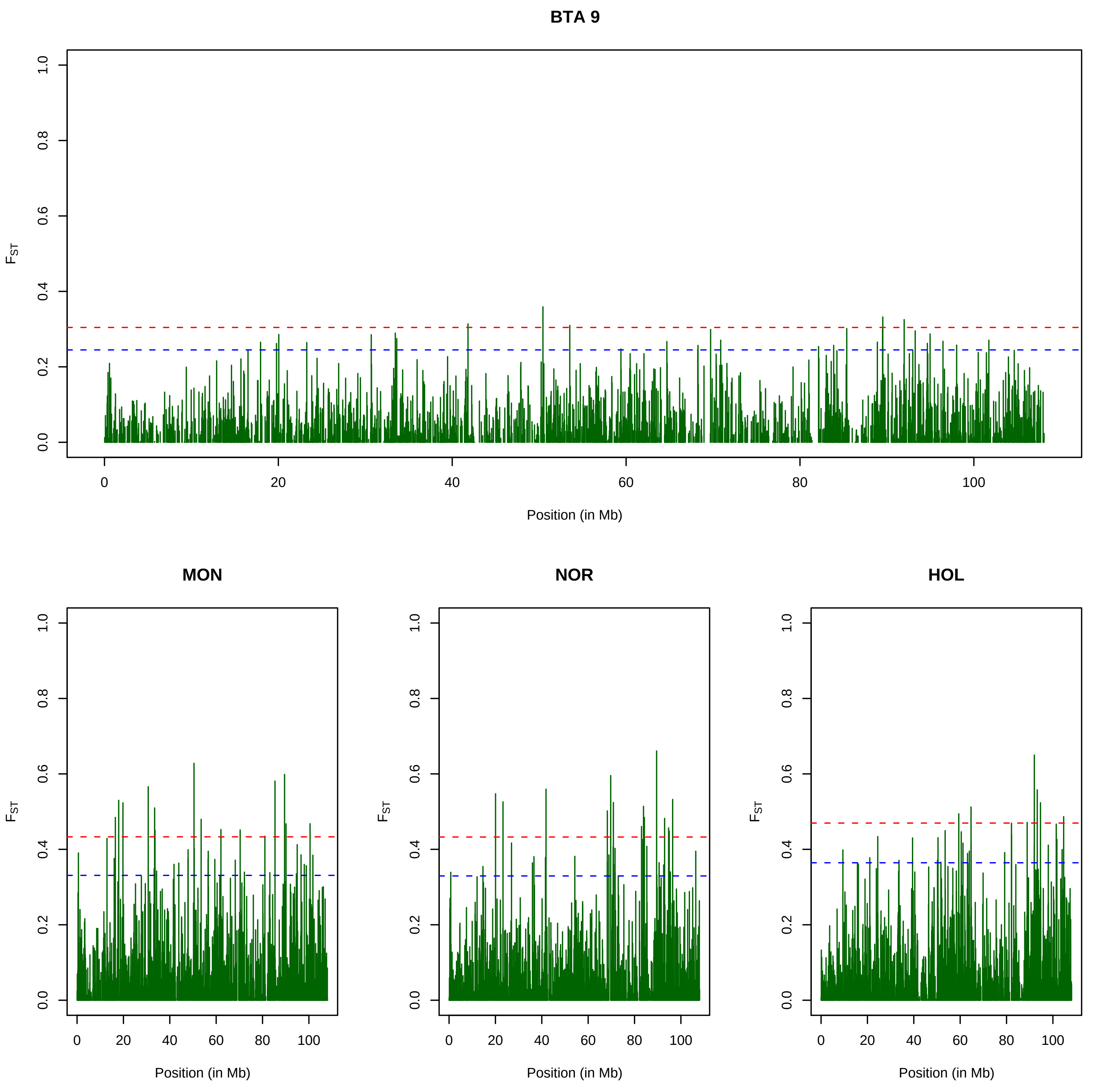
BTA 9
MON
NOR
HOL
F_{ST}
Position (in Mb)

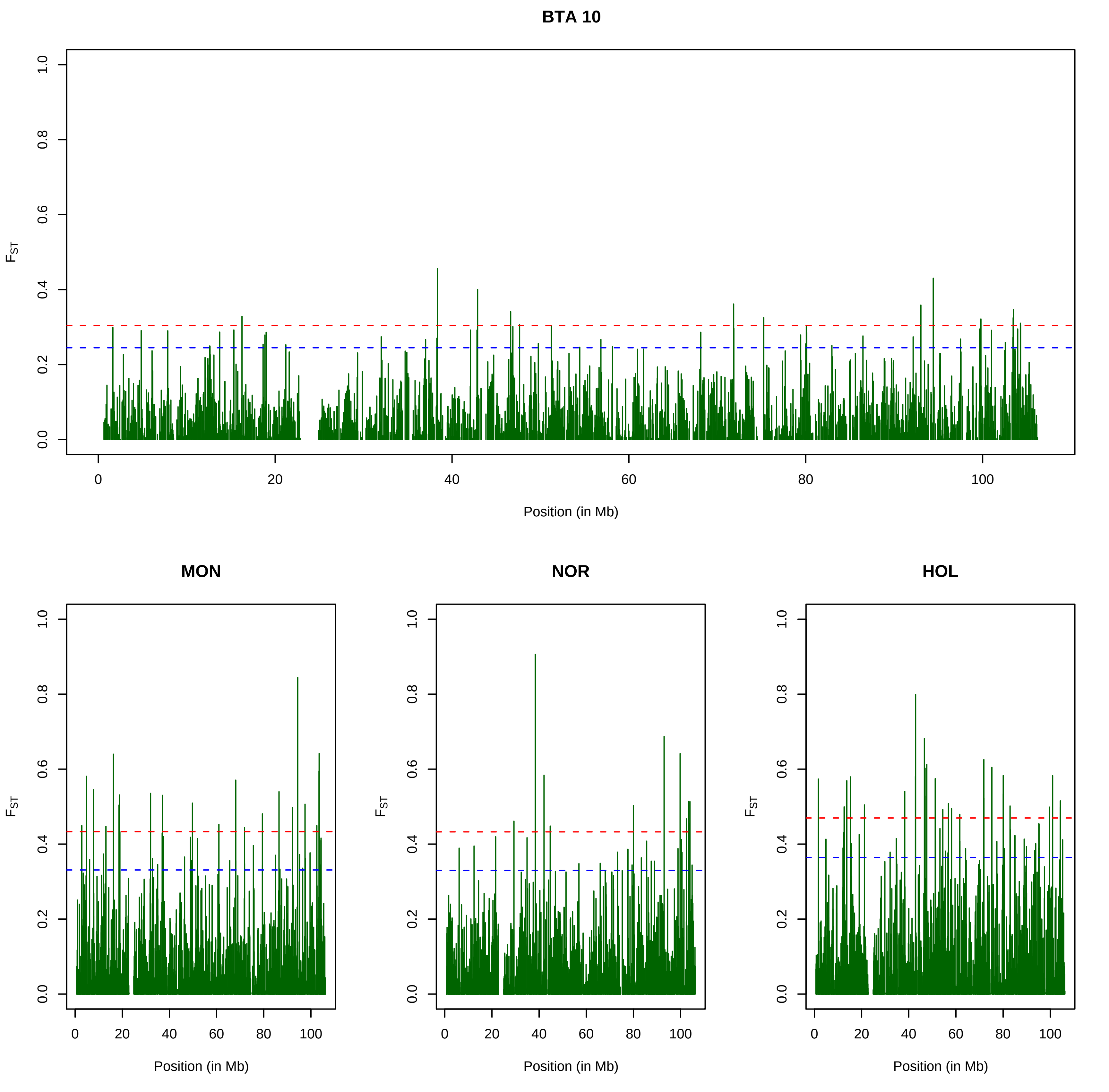

BTA 10
F_ST
Position (in Mb)
MON
NOR
HOL
0.0
0.2
0.4
0.6
0.8
1.0
0
20
40
60
80
100

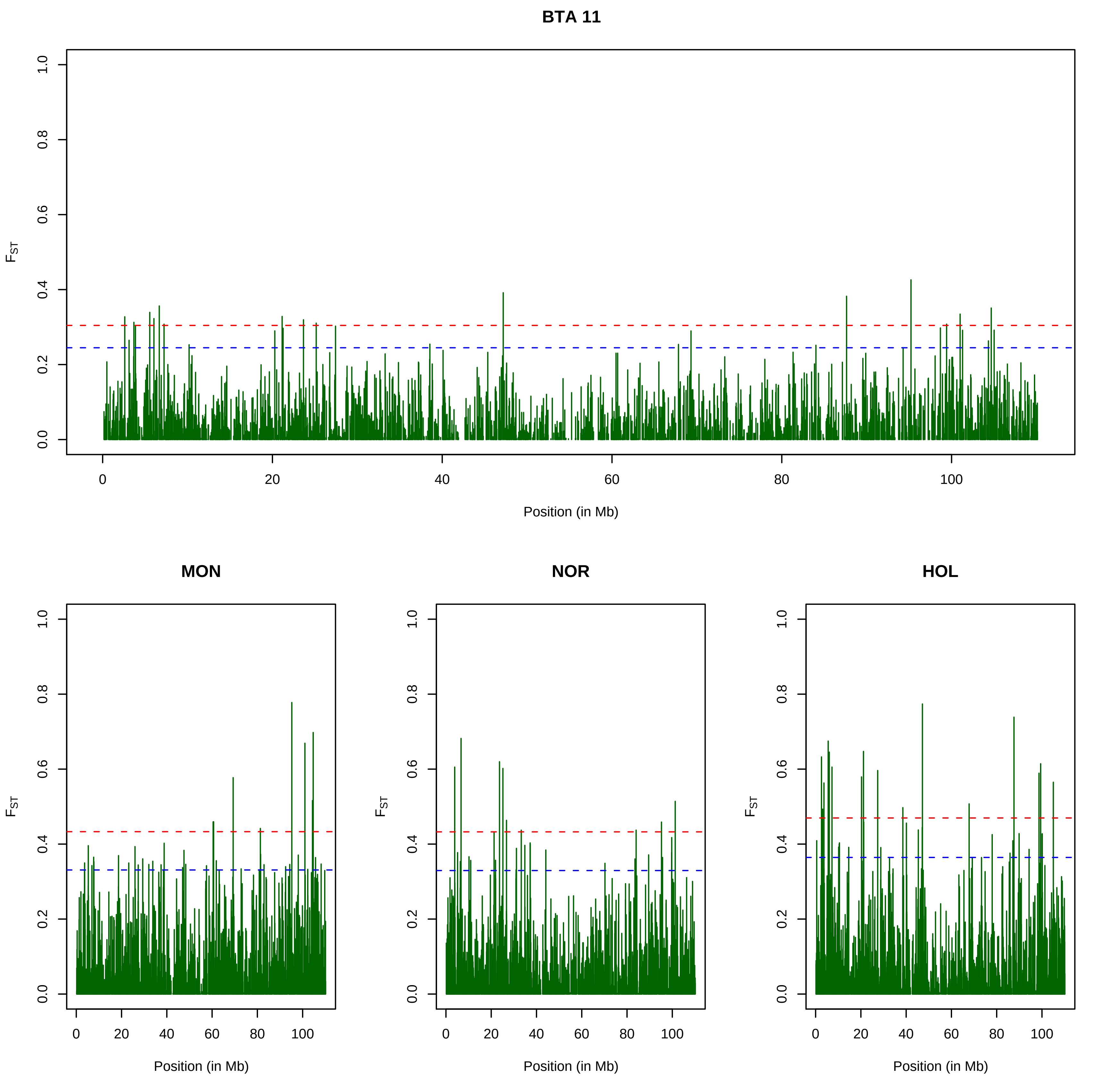
BTA 11
MON
NOR
HOL
FST
Position (in Mb)

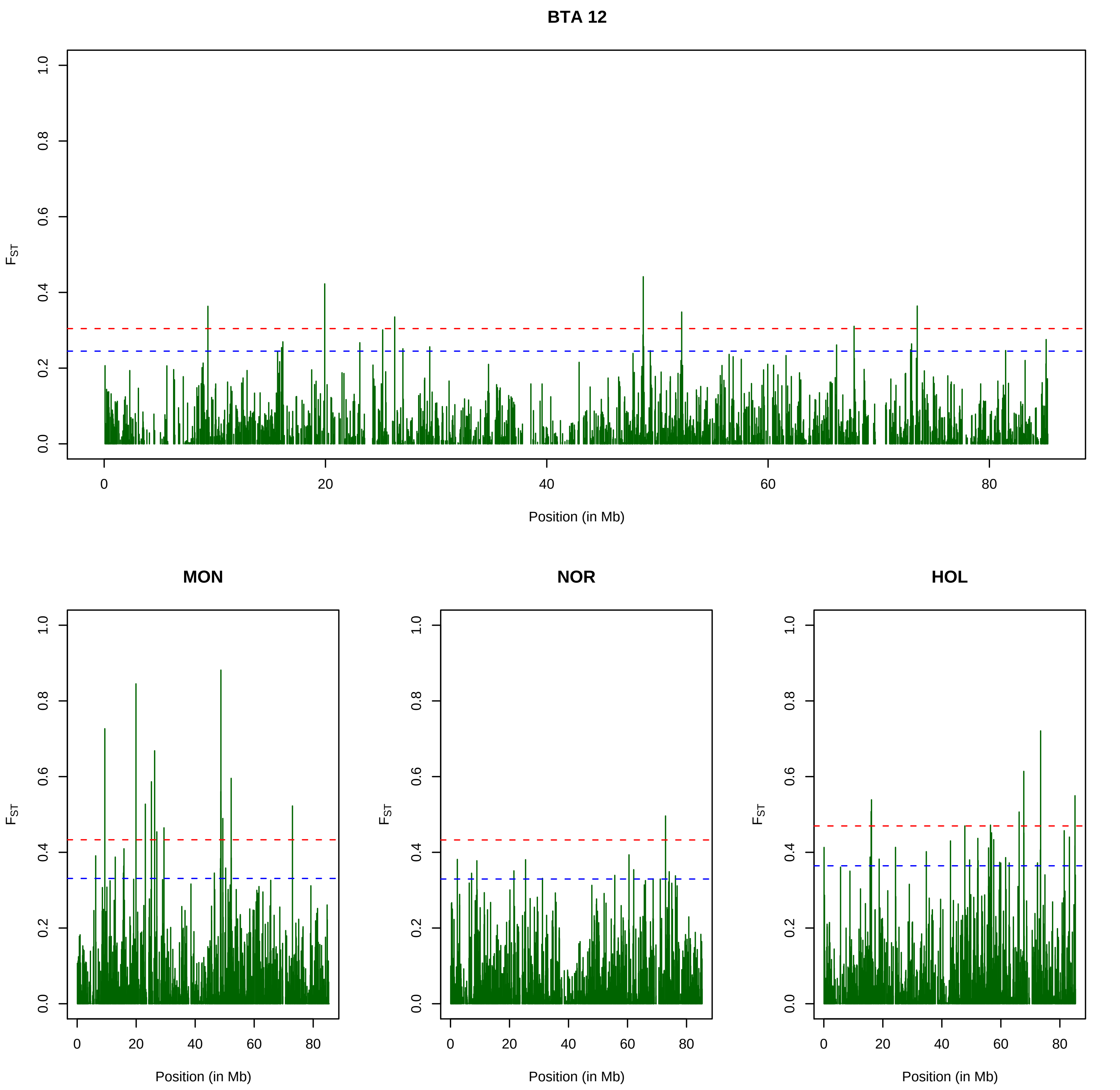

BTA 12
MON
NOR
HOL
F_{ST}
Position (in Mb)

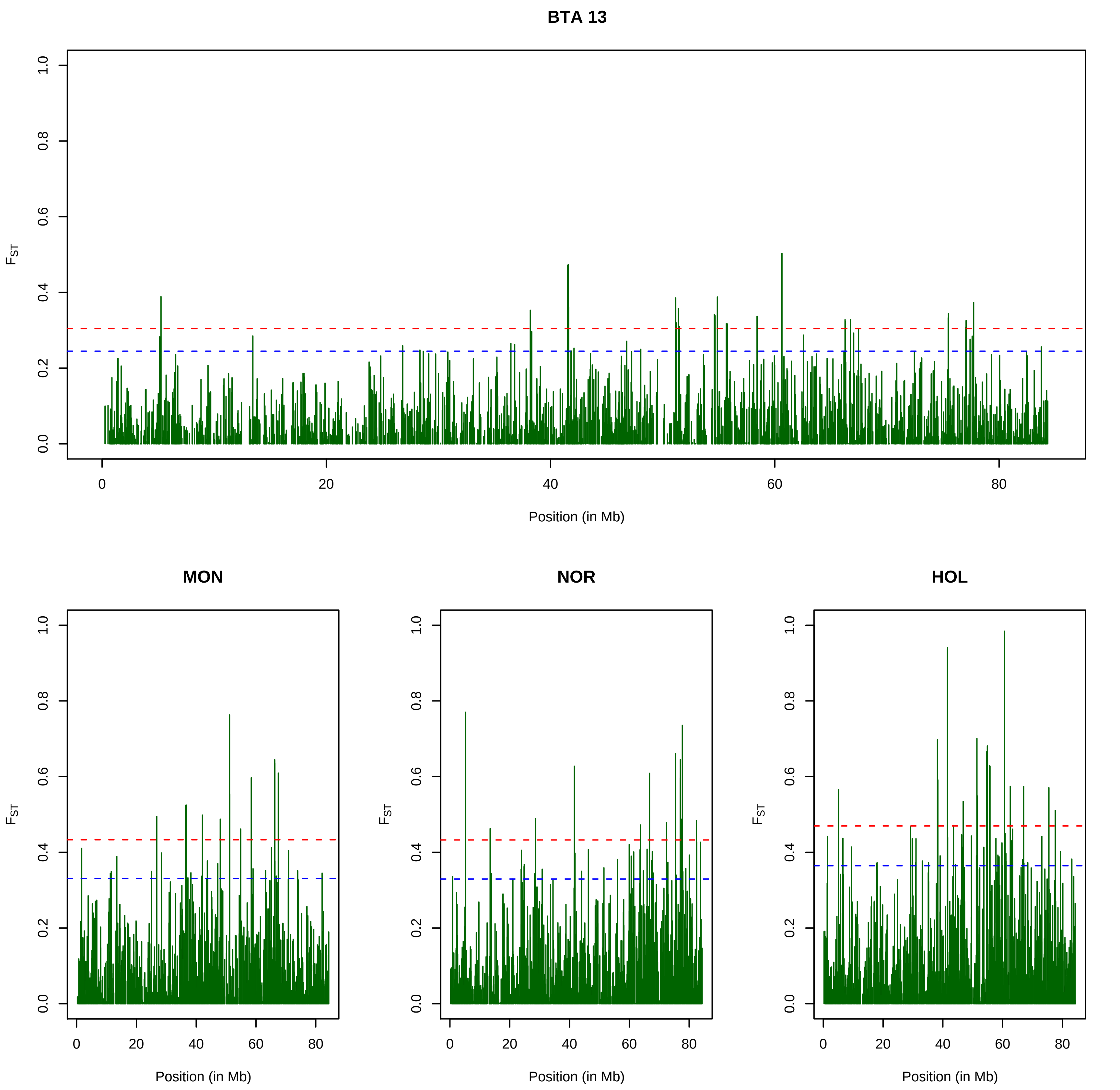

BTA 13
MON
NOR
HOL
F_ST
Position (in Mb)

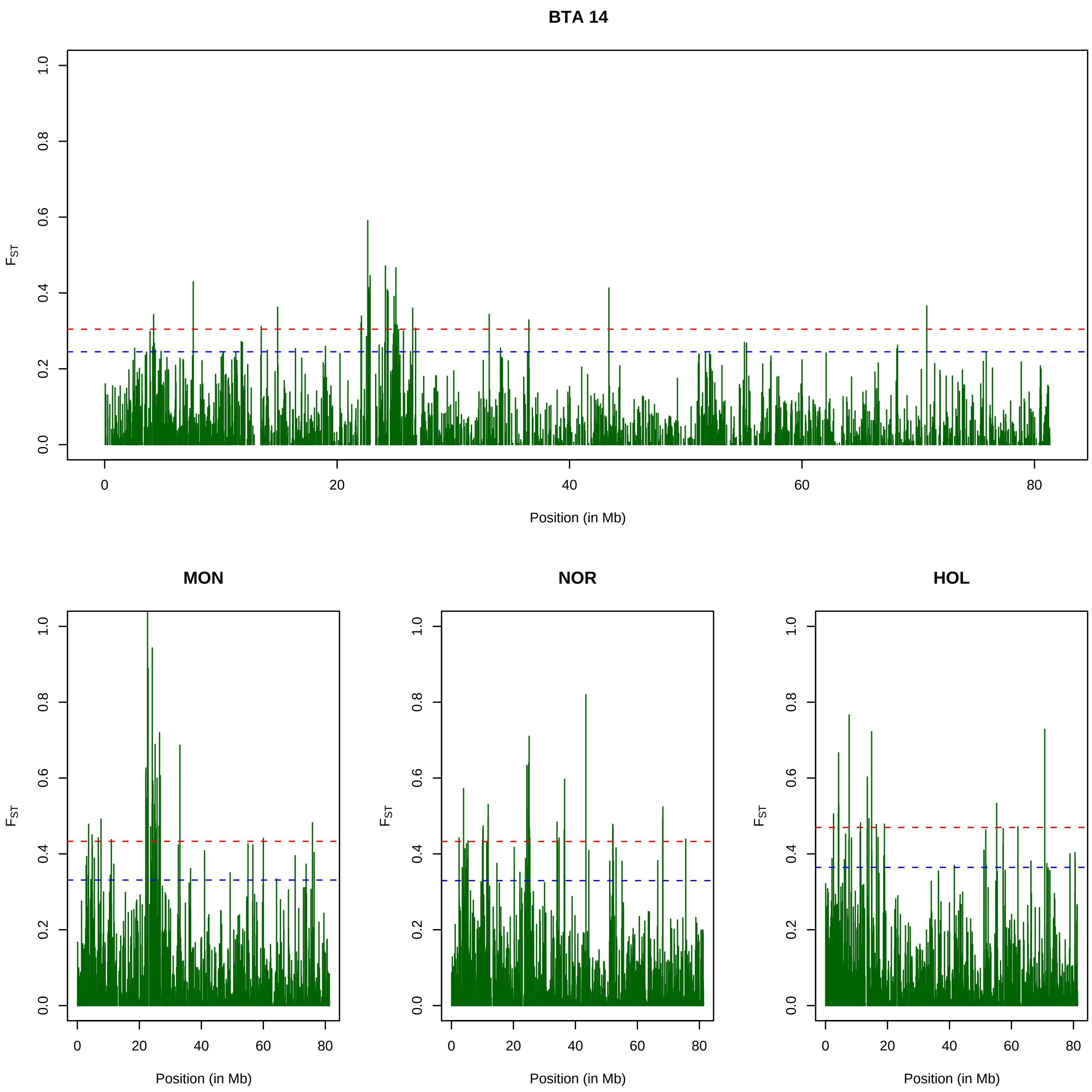

BTA 14
MON
NOR
HOL
F_ST
Position (in Mb)
0.0
0.2
0.4
0.6
0.8
1.0
0
20
40
60
80

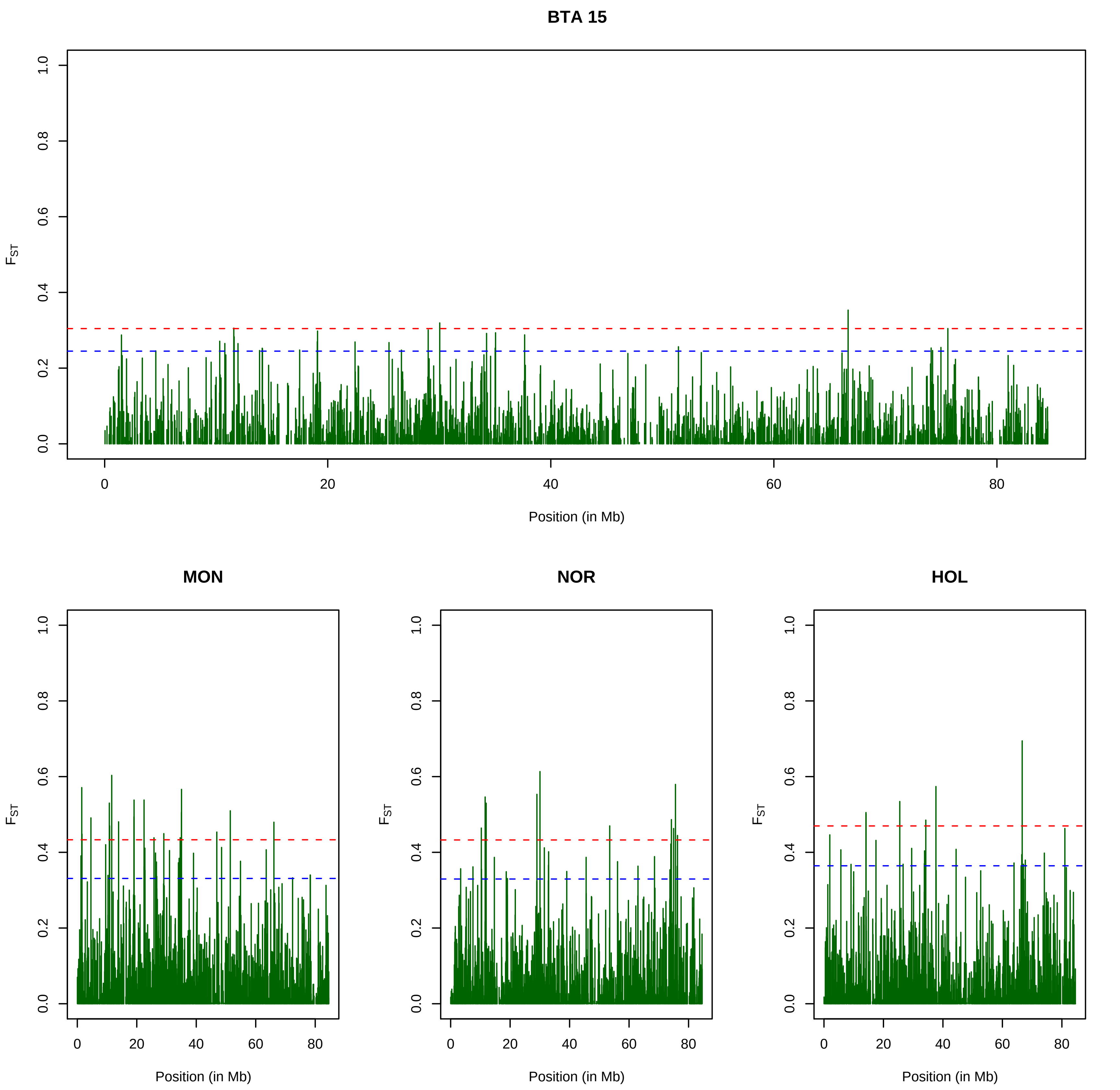

BTA 15
MON
NOR
HOL
F_{ST}
Position (in Mb)

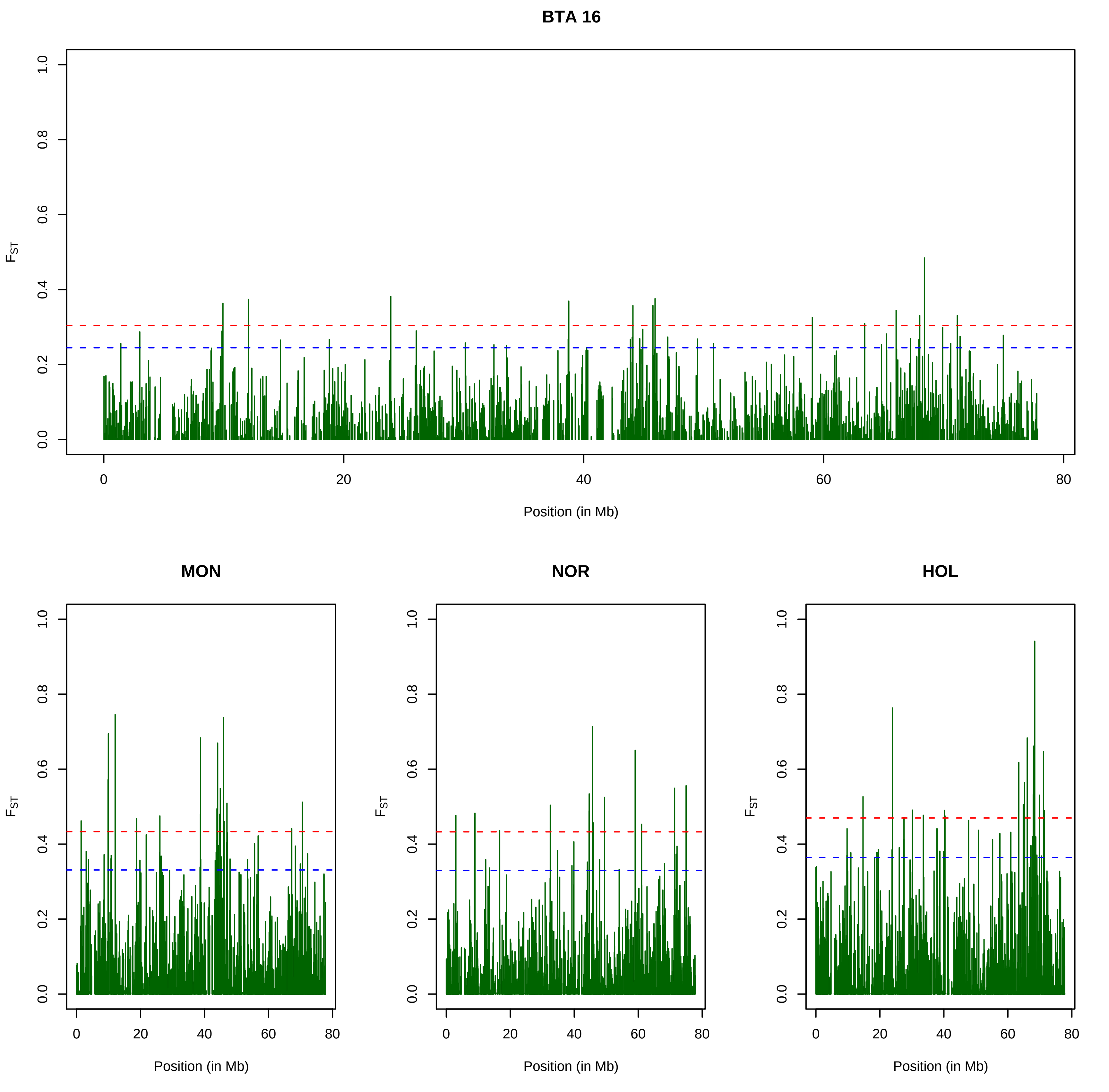

BTA 16
MON
NOR
HOL
F_{ST}
Position (in Mb)

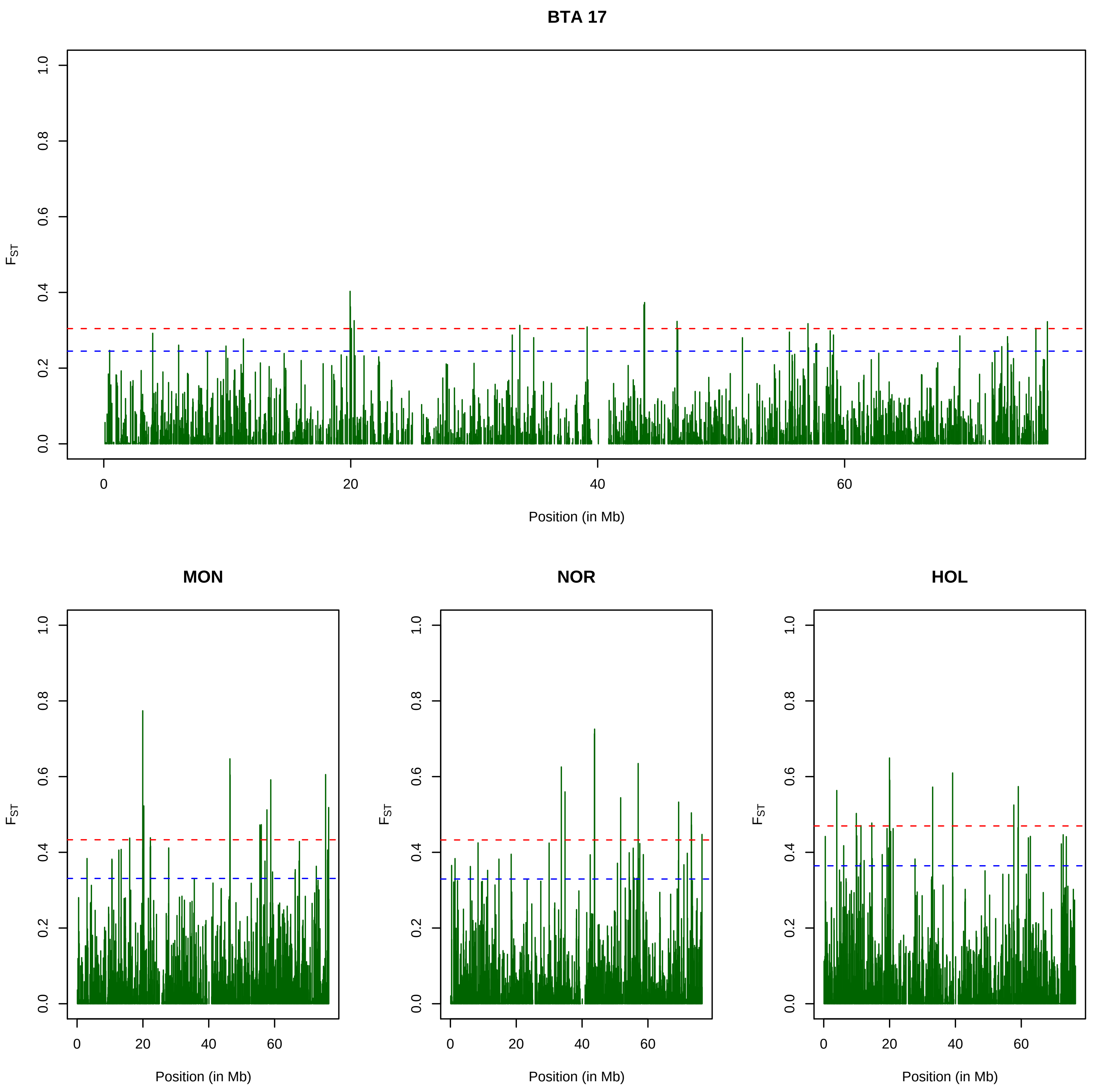

BTA 17
MON
NOR
HOL
F_{ST}
0.0
0.2
0.4
0.6
0.8
1.0
0
20
40
60
Position (in Mb)

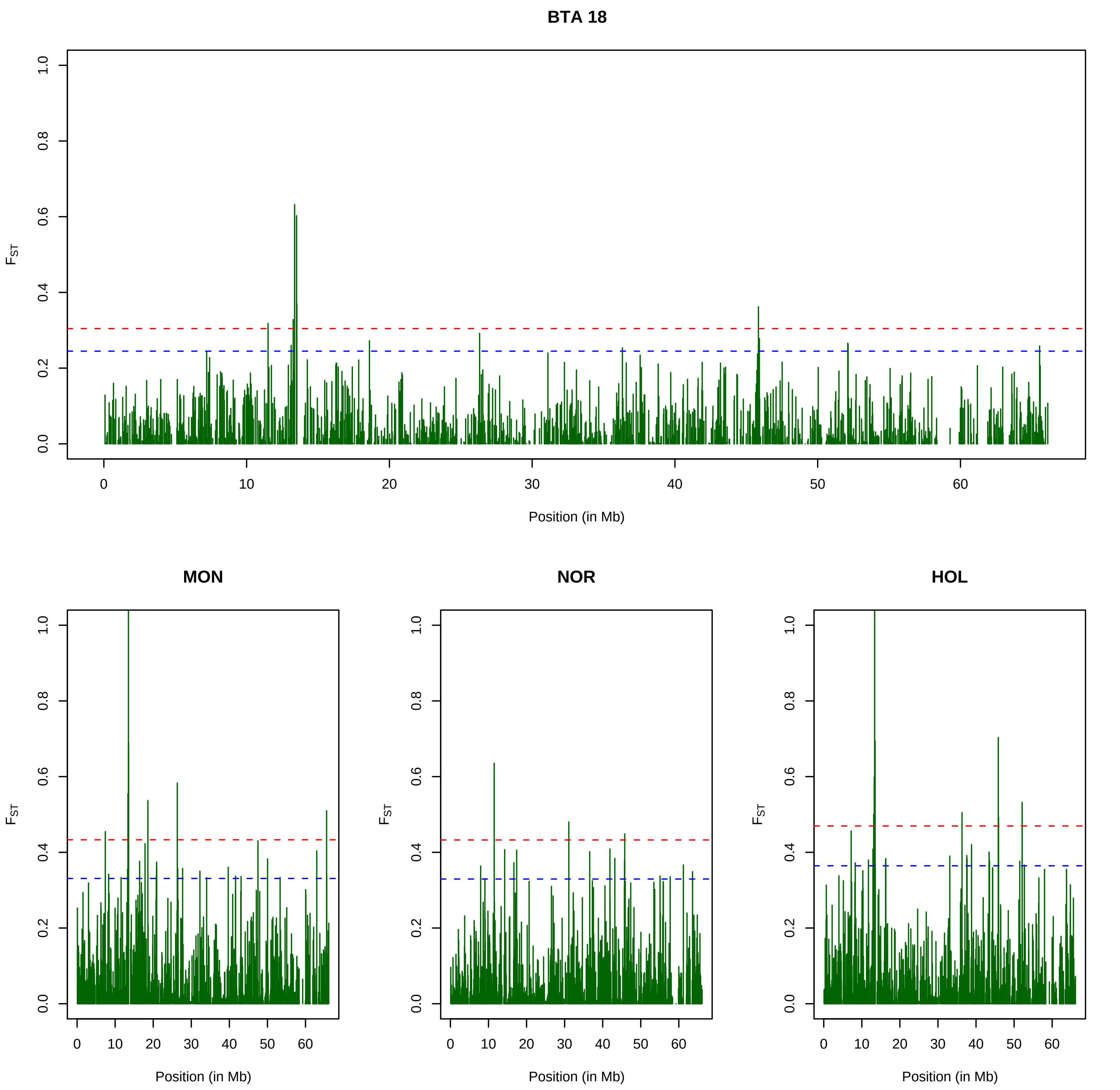

BTA 18
FST
0.0
0.2
0.4
0.6
0.8
1.0
0
10
20
30
40
50
60
Position (in Mb)
MON
NOR
HOL

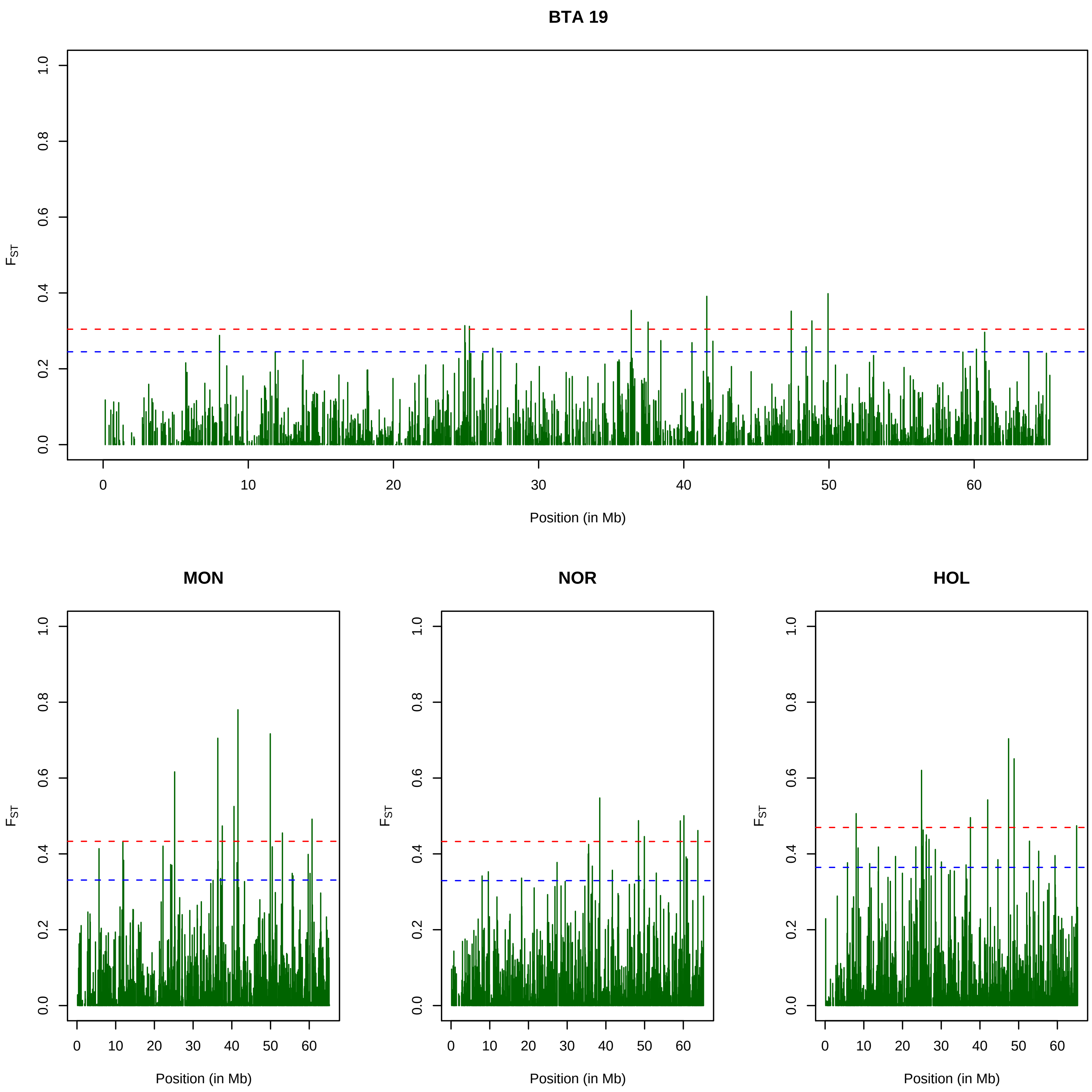

BTA 19
FST
Position (in Mb)
MON
NOR
HOL
0.0
0.2
0.4
0.6
0.8
1.0
0
10
20
30
40
50
60

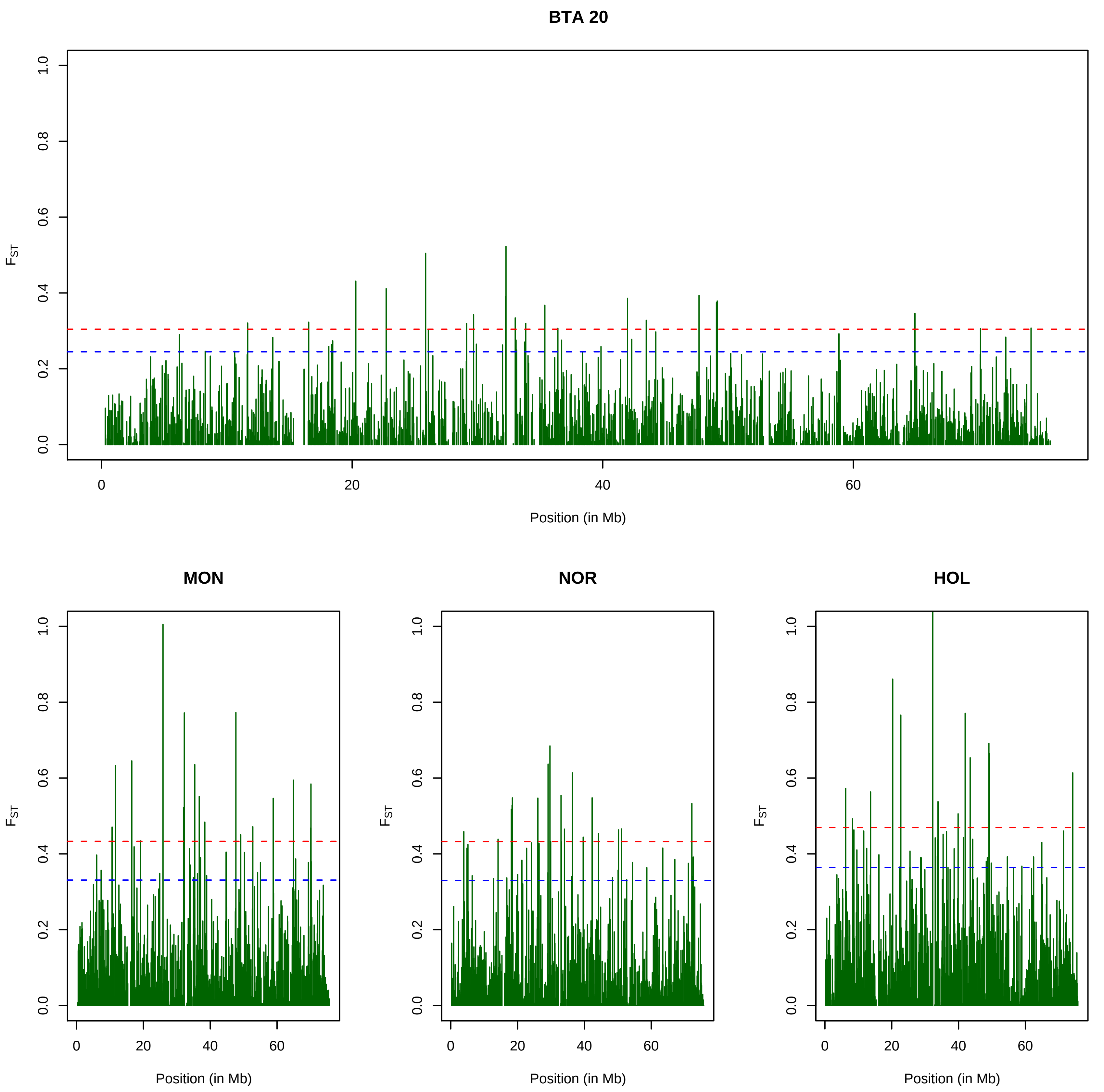
BTA 20
MON
NOR
HOL
F_{ST}
Position (in Mb)
0.0
0.2
0.4
0.6
0.8
1.0
0
20
40
60

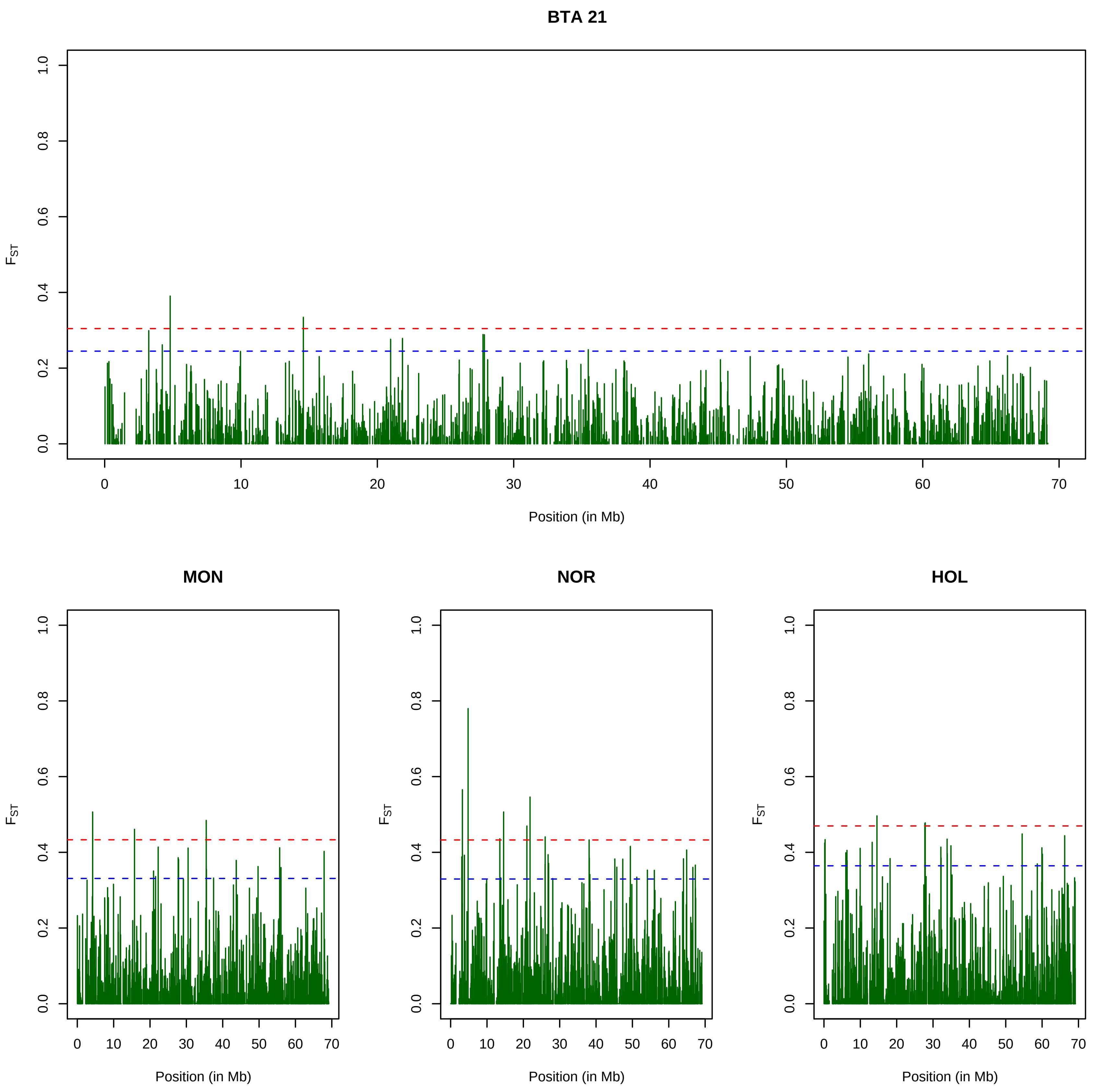

BTA 21
F_ST
Position (in Mb)
MON
NOR
HOL

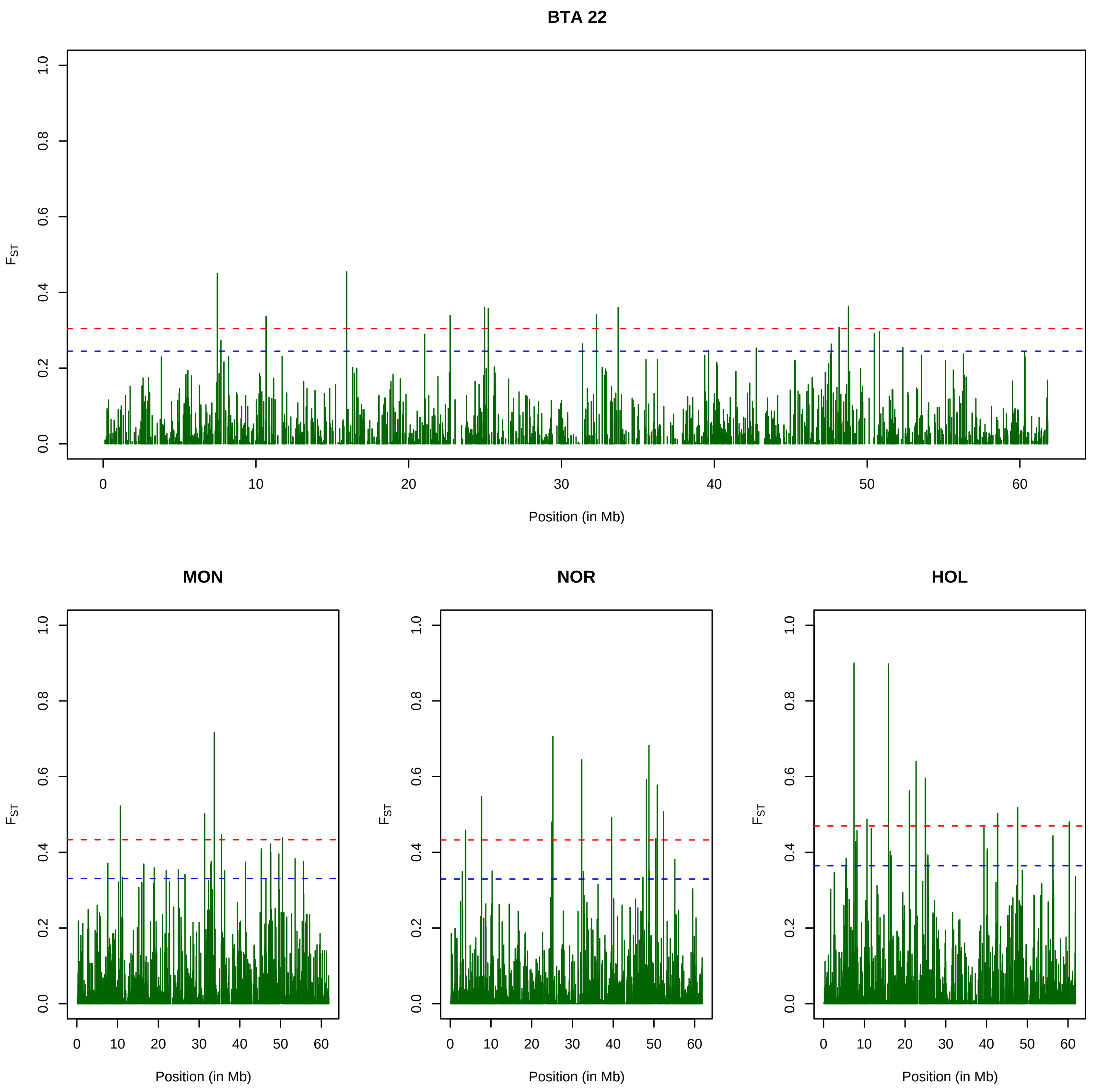
BTA 22
F_ST
0.0
0.2
0.4
0.6
0.8
1.0
0
10
20
30
40
50
60
Position (in Mb)
MON
NOR
HOL

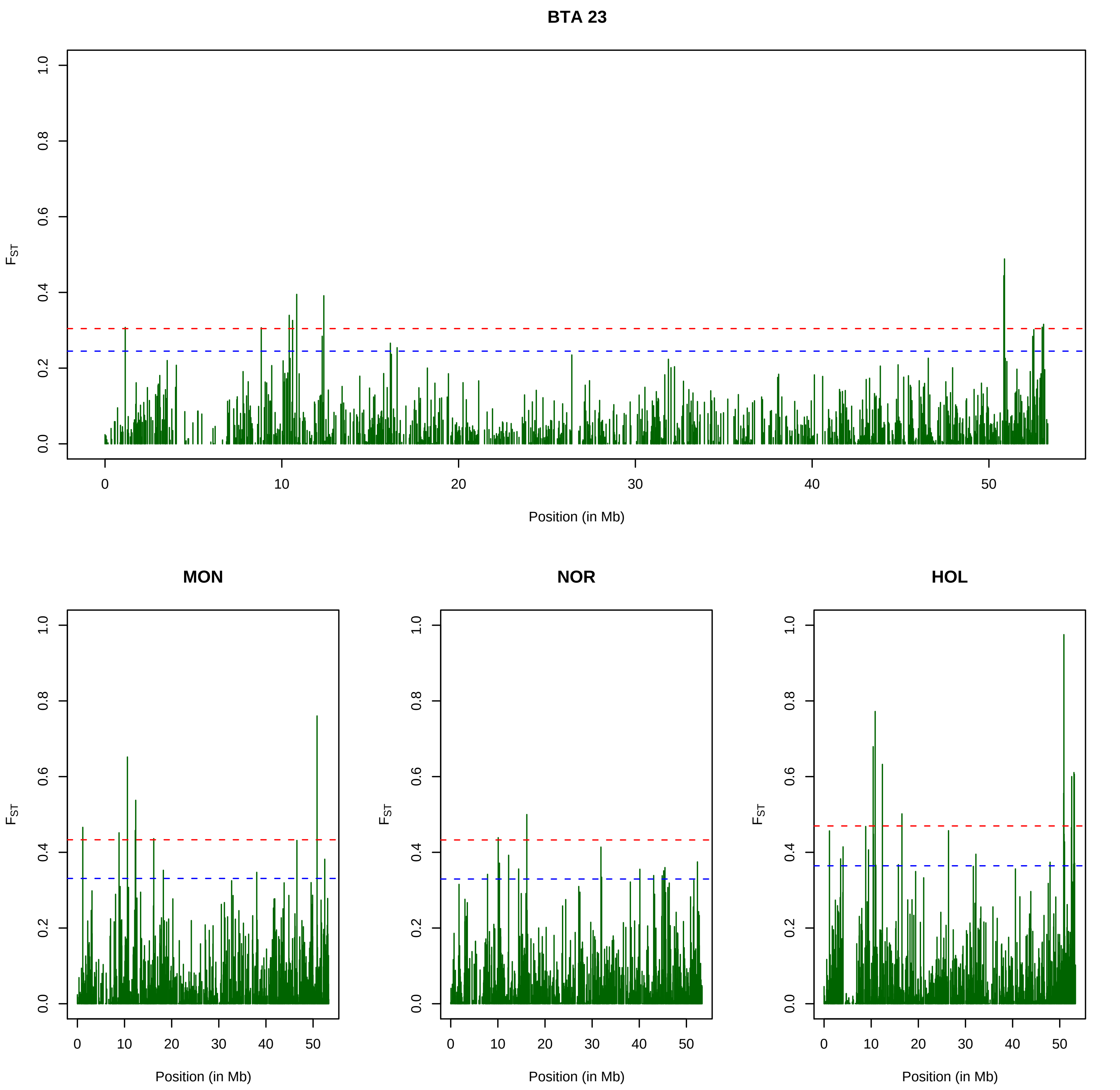

BTA 23
F_ST
Position (in Mb)
MON
NOR
HOL

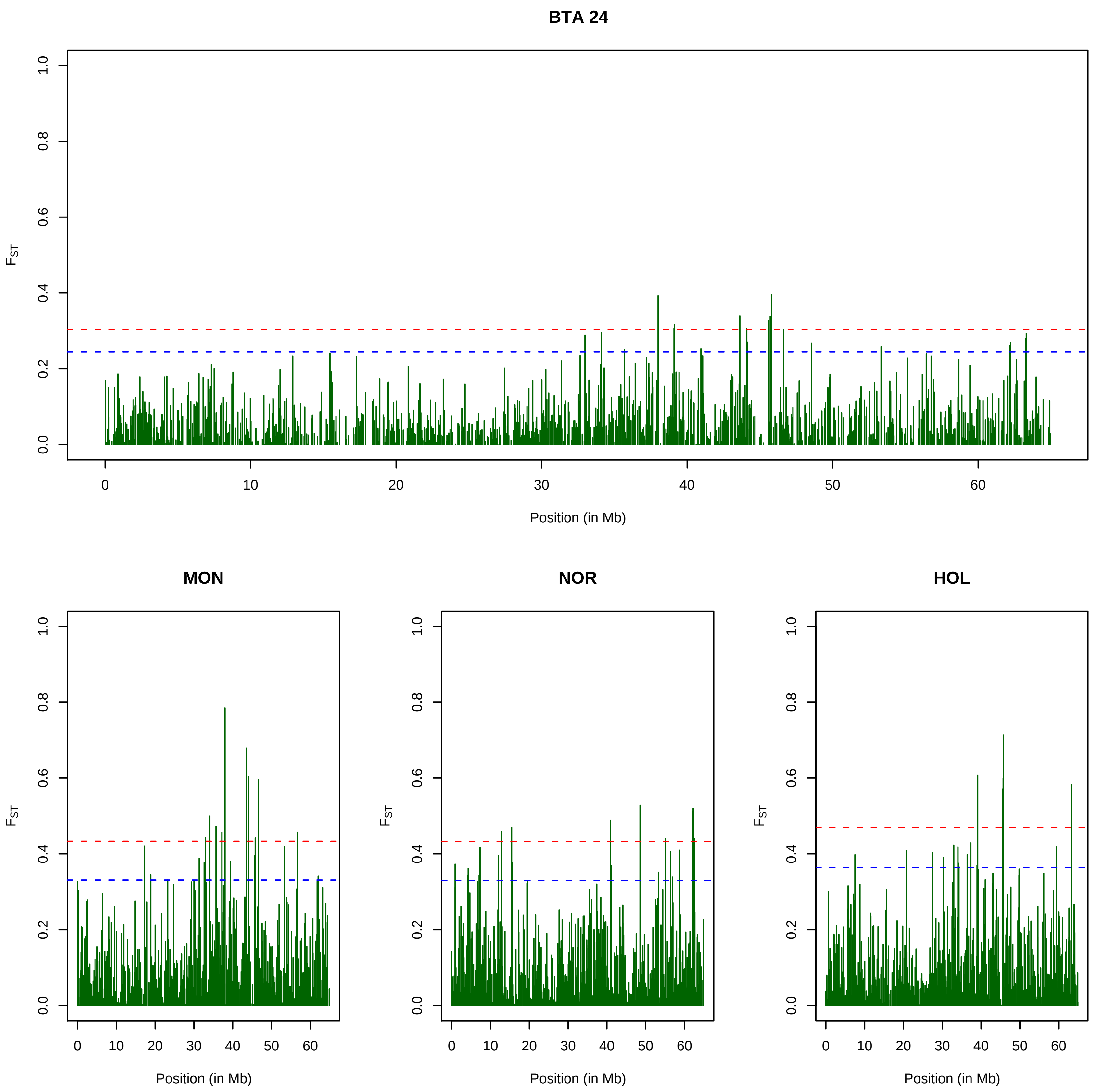

BTA 24
MON
NOR
HOL
F_ST
Position (in Mb)

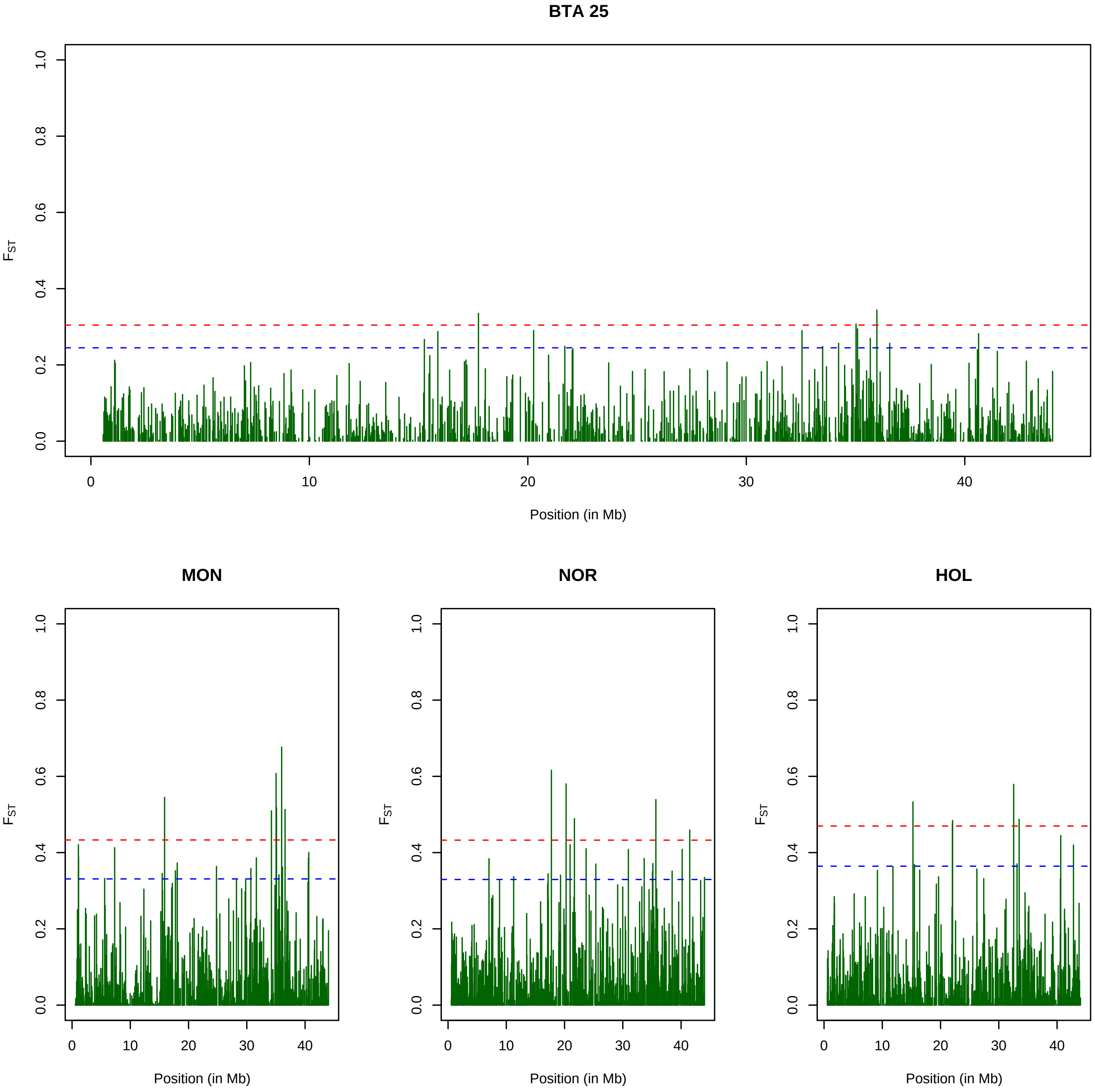

BTA 25
FST
1.0
0.8
0.6
0.4
0.2
0.0
0
10
20
30
40
Position (in Mb)
MON
NOR
HOL

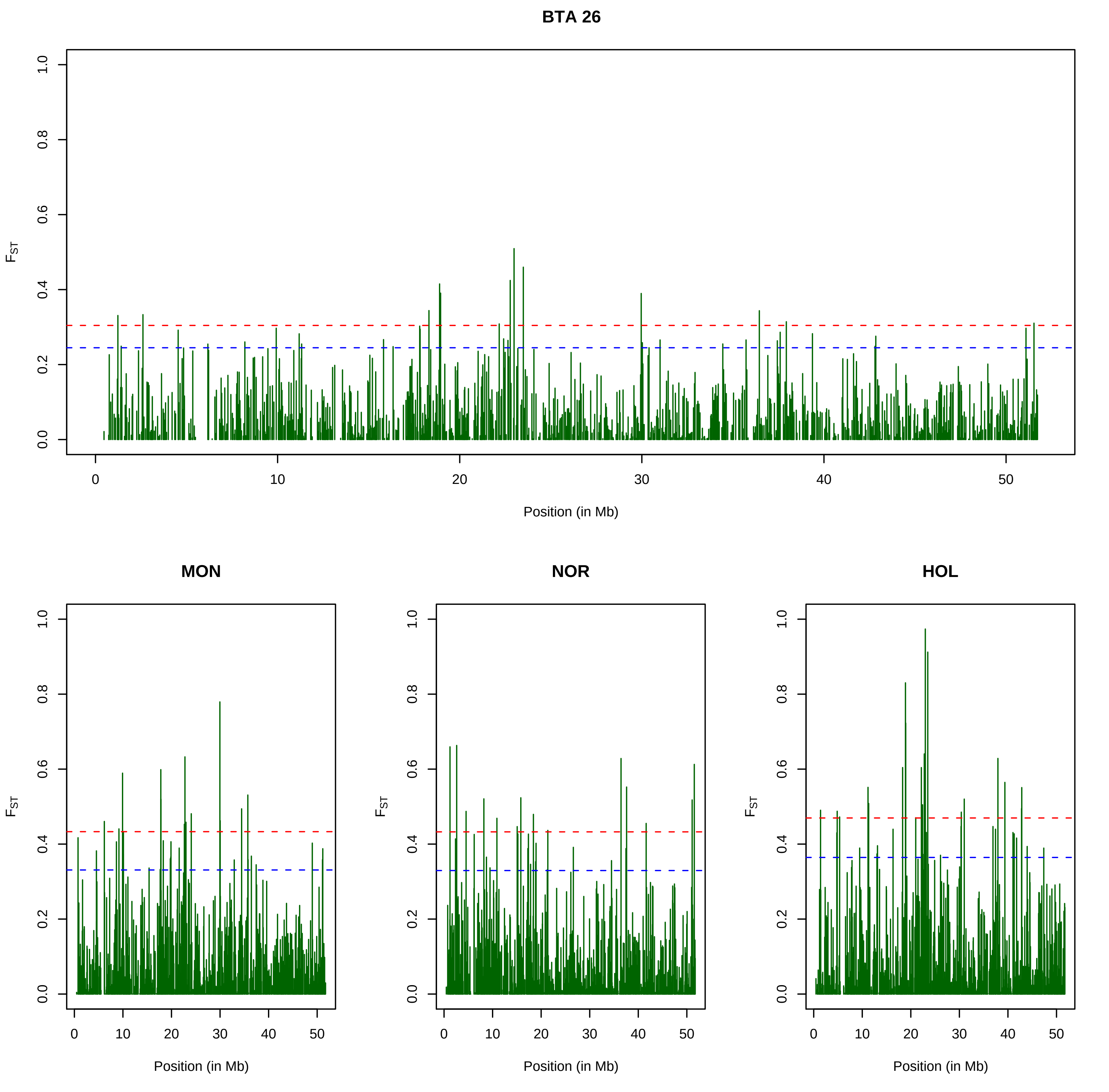

BTA 26
MON
NOR
HOL
F_ST
Position (in Mb)

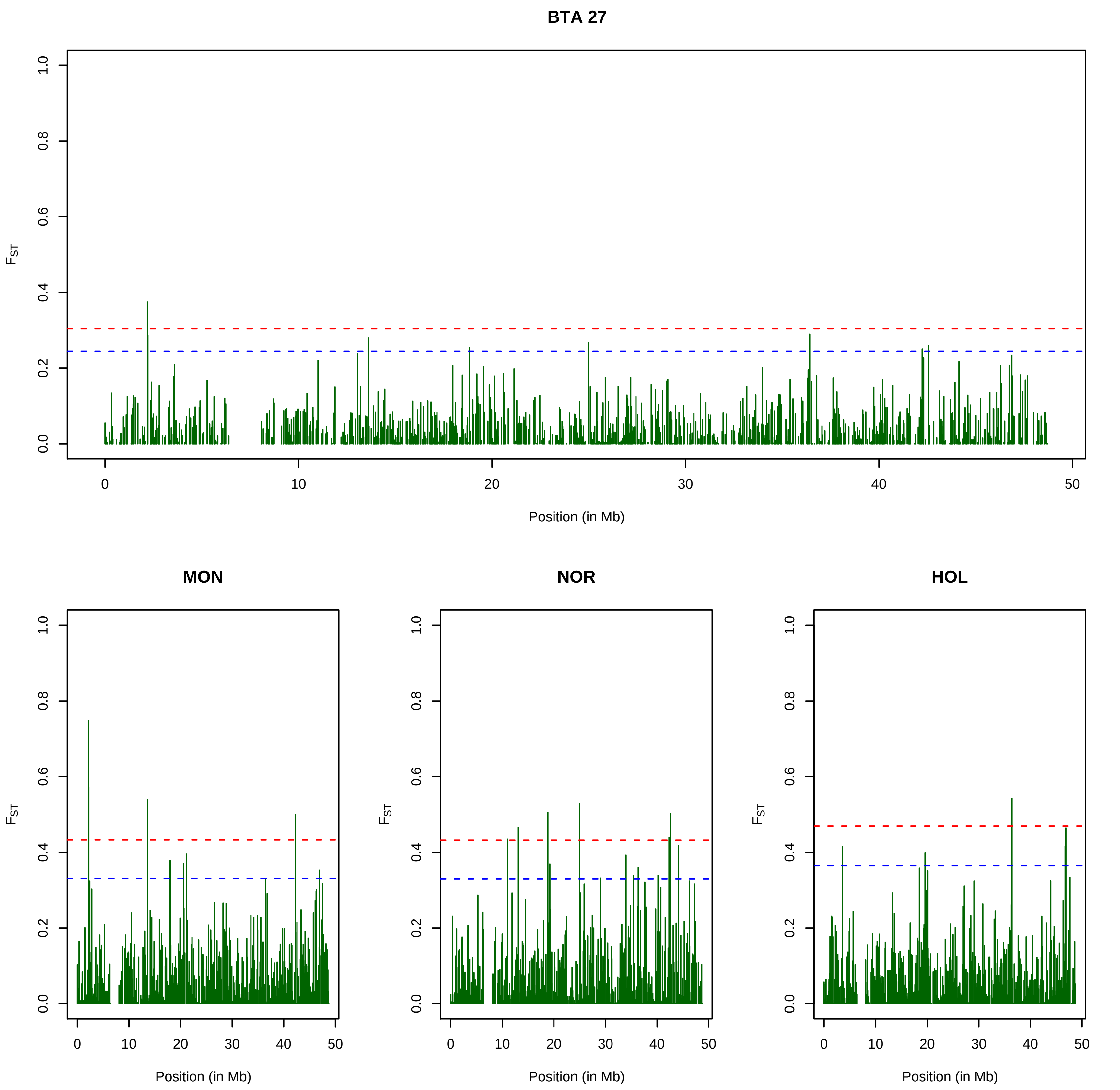
BTA 27
F_ST
0.0
0.2
0.4
0.6
0.8
1.0
0
10
20
30
40
50
Position (in Mb)
MON
NOR
HOL
F_ST
Position (in Mb)
F_ST
Position (in Mb)
F_ST
Position (in Mb)

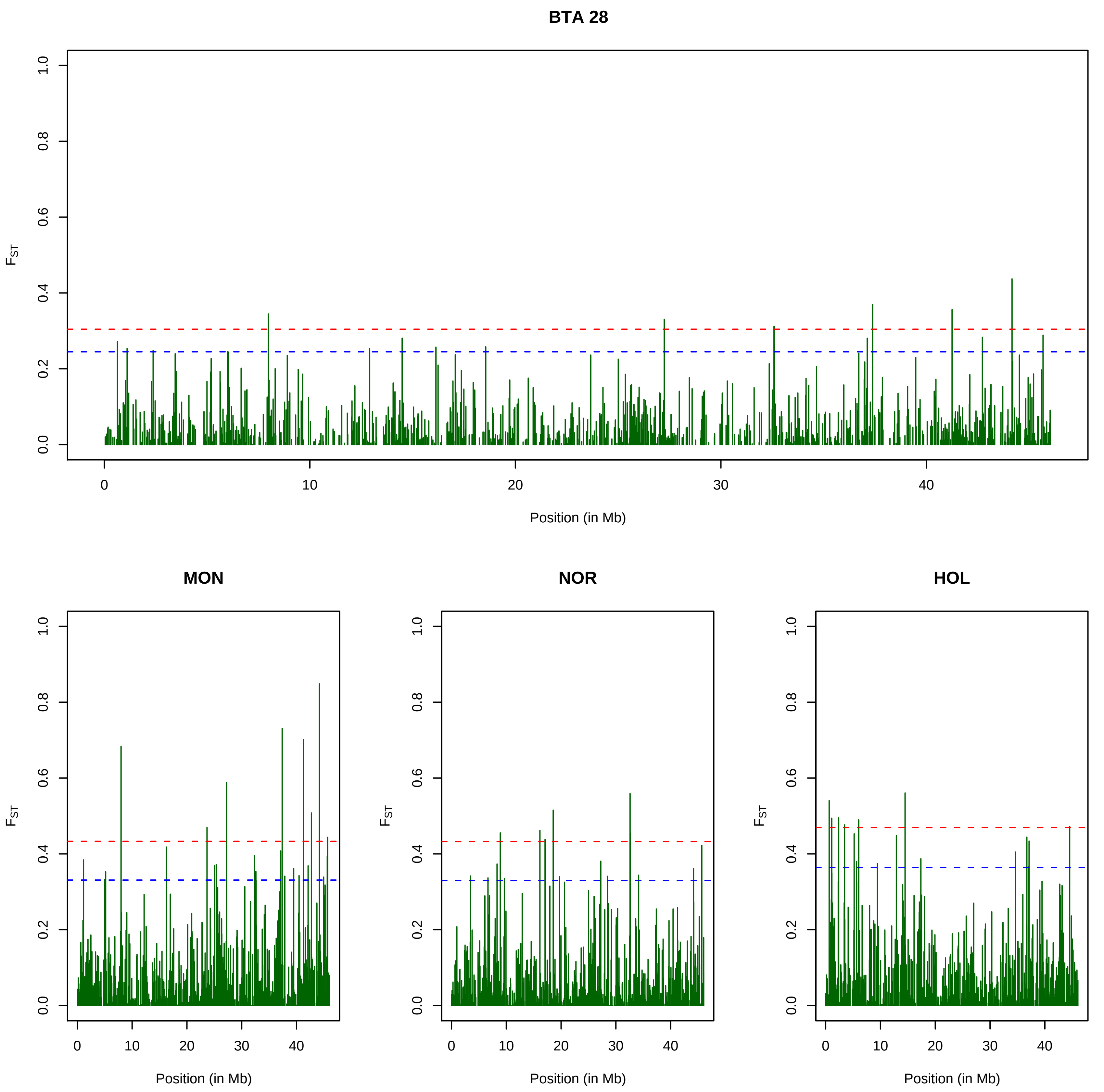
BTA 28
FST
0.0
0.2
0.4
0.6
0.8
1.0
0
10
20
30
40
Position (in Mb)
MON
NOR
HOL

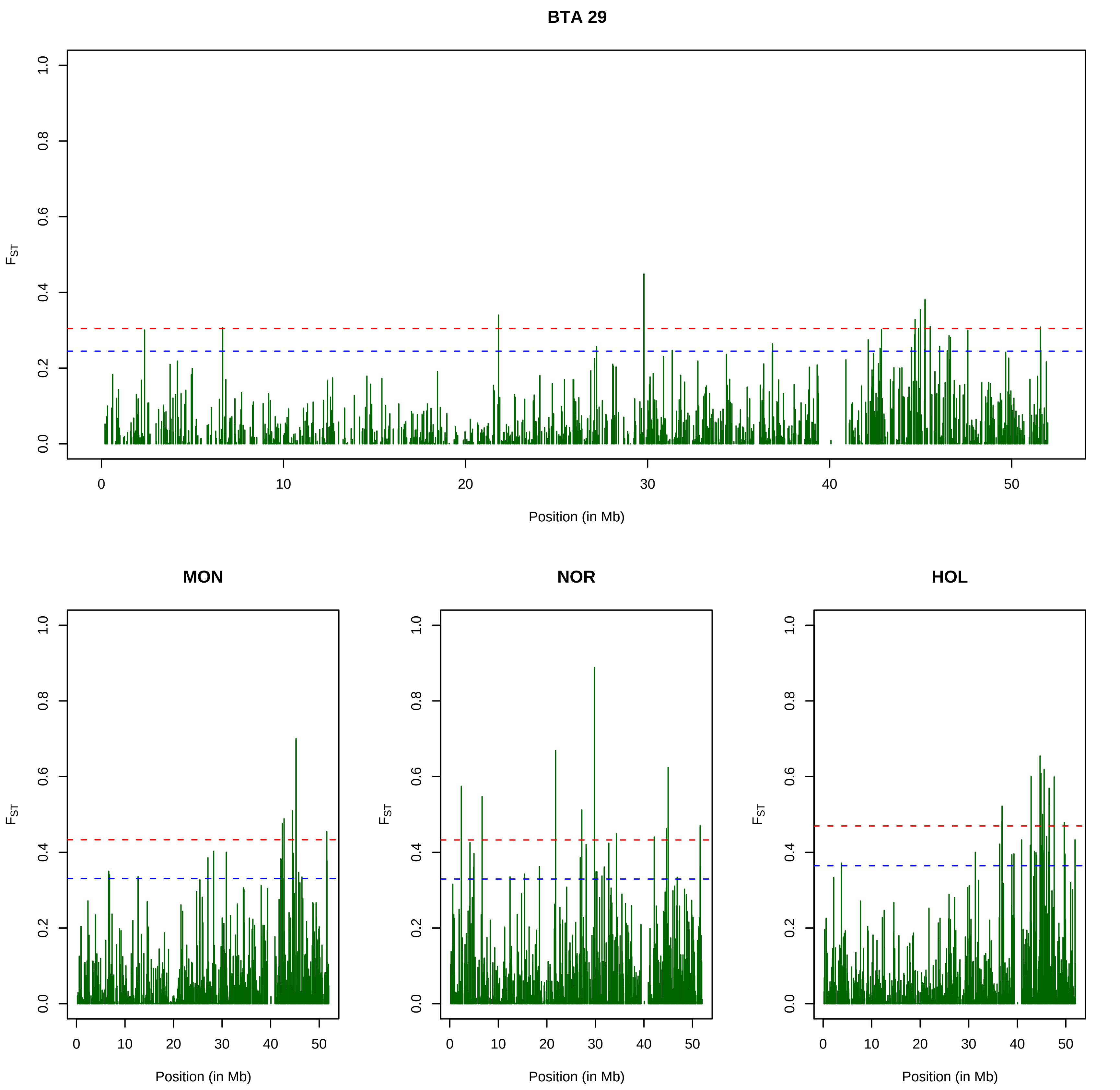
BTA 29
F_ST
0.0
0.2
0.4
0.6
0.8
1.0
0
10
20
30
40
50
Position (in Mb)
MON
NOR
HOL
