# Supplementary figures and images for: The Genome Response to Artificial Selection: A Case Study in Dairy Cattle
Source: PLoS One. 2009 Aug 12;4(8):e6595. doi: 10.1371/journal.pone.0006595 (PMC2722727; doi:10.1371/journal.pone.0006595)

## Observed

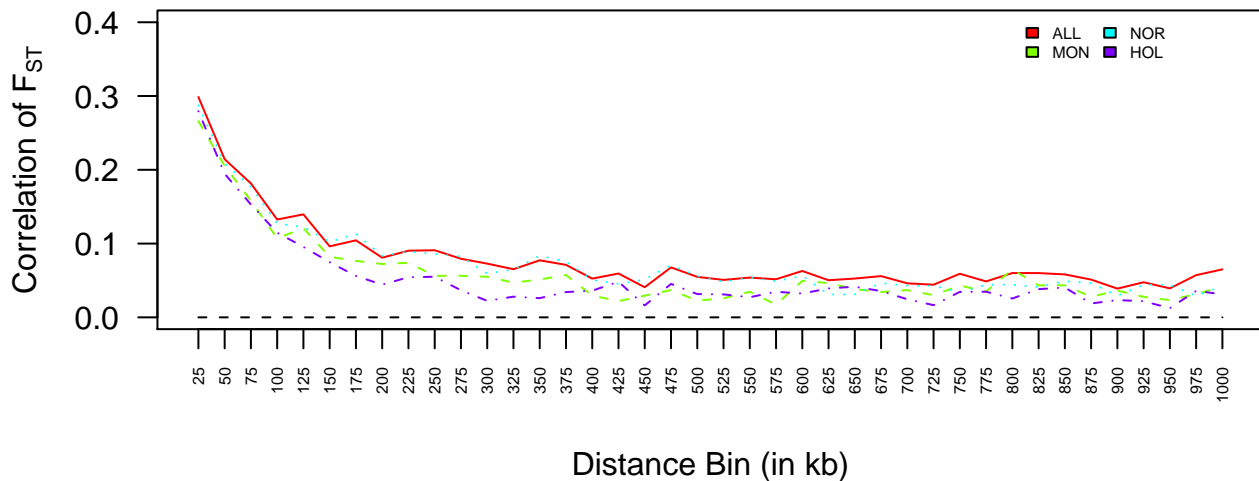

## Simulated

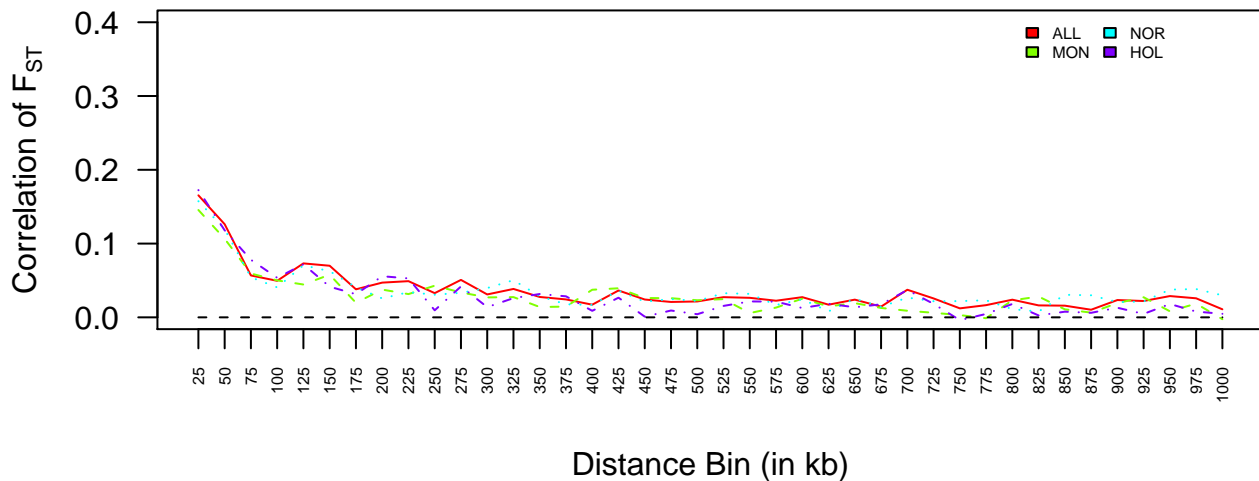

Supplement: Figure S2 — Correlation of FST (across and within each of the three breeds) for pairs of markers as a function of physical distances in the real (upper panel) and simulated (lower panel) data sets. (0.02 MB PDF) [file pone.0006595.s005.pdf]

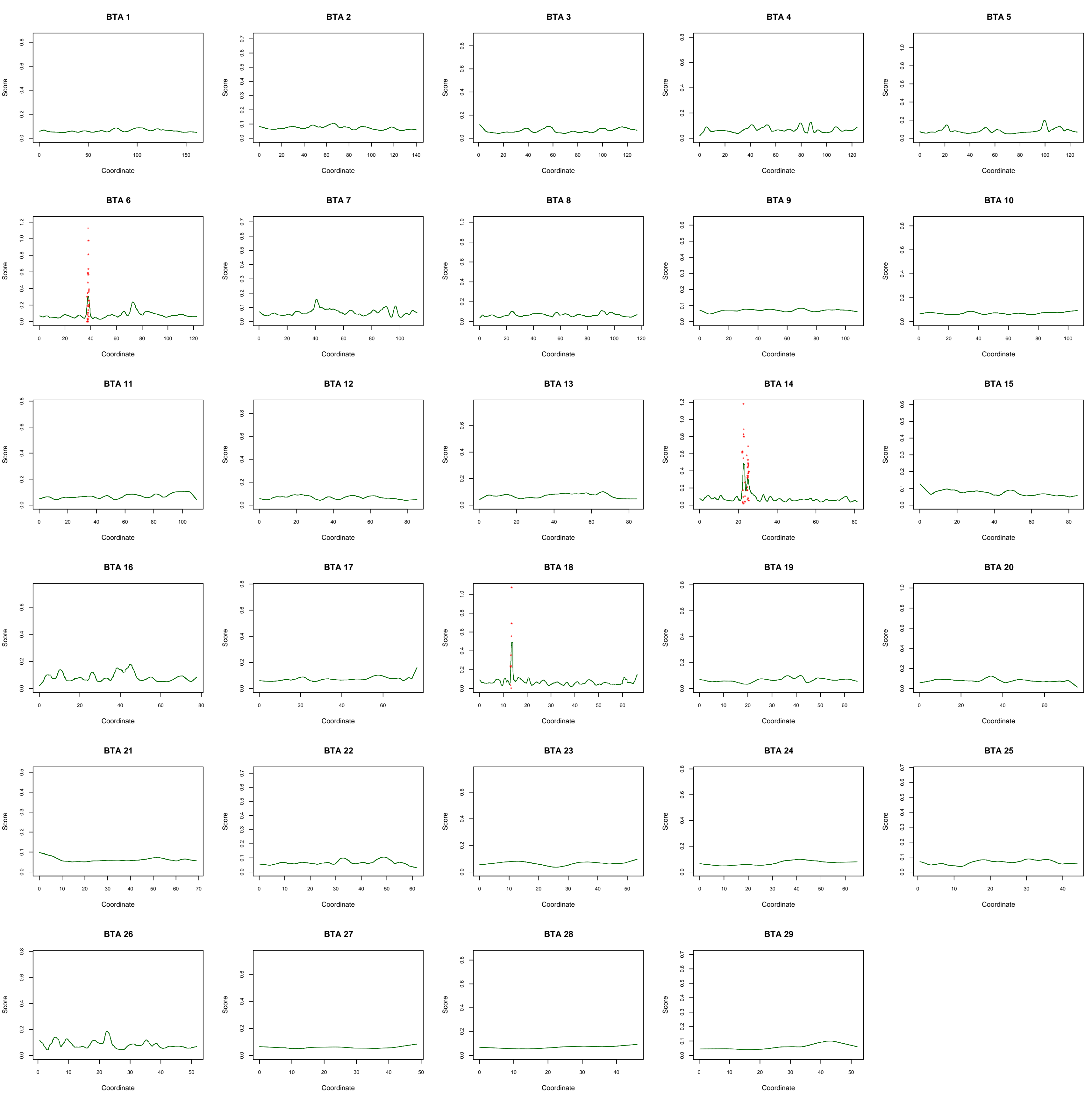

Supplement: Figure S3 — Whole genome map of regions under selection based on the FST within MON. For each of the 29 bovine autosomes, the smoothed FST is plotted against the chromosomal position (green line). For significant positions (q-value<0.05), non smoothed SNP FST are indicated by a red star. (0.22 MB ZIP) [file pone.0006595.s006.zip › FigureS3.pdf]

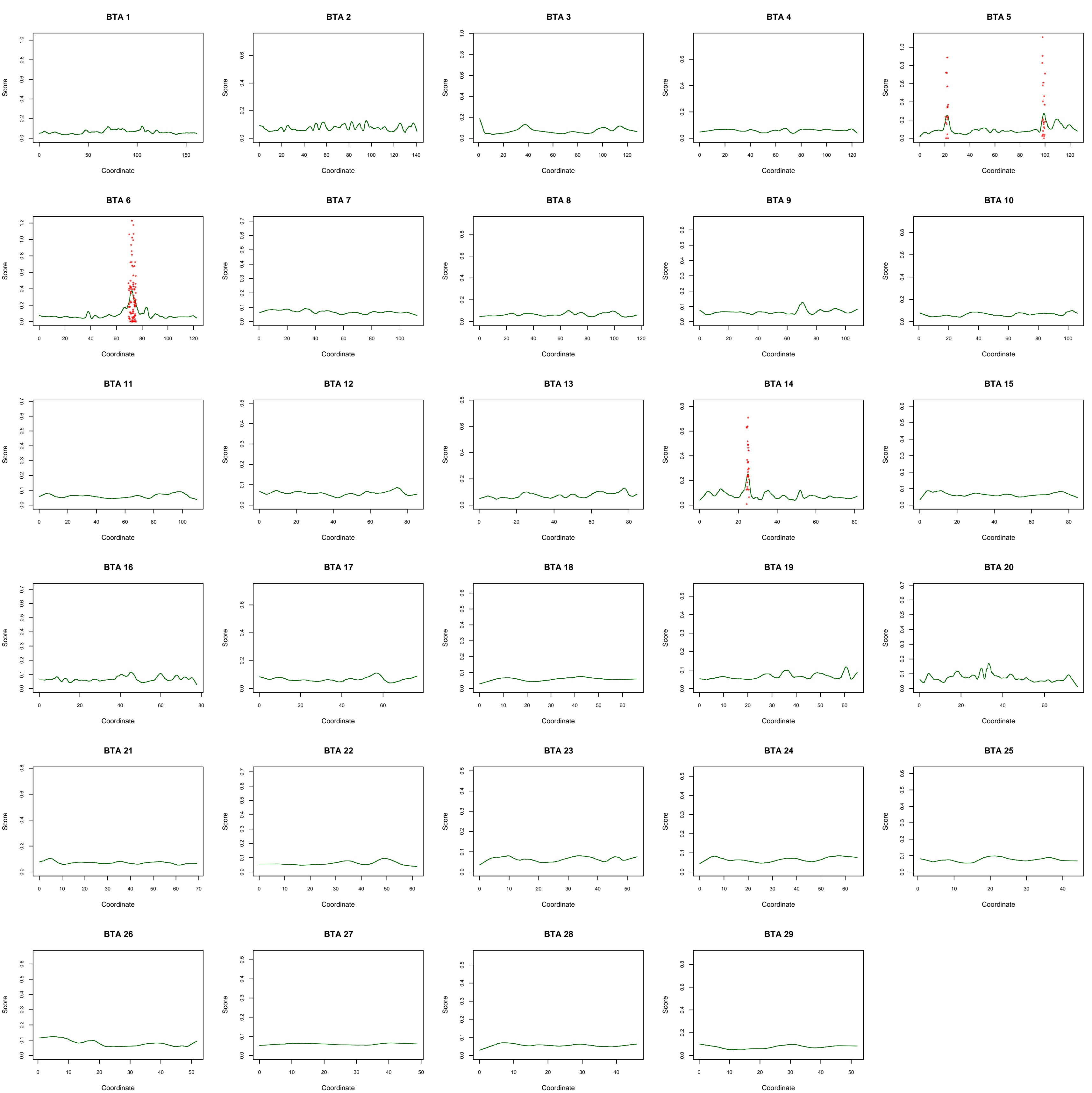

Supplement: Figure S4 — Whole genome map of regions under selection based on the FST within NOR. For each of the 29 bovine autosomes, the smoothed FST is plotted against the chromosomal position (green line). For significant positions (q-value<0.05), non smoothed SNP FST are indicated by a red star. (0.22 MB ZIP) [file pone.0006595.s007.zip › FigureS4.pdf]

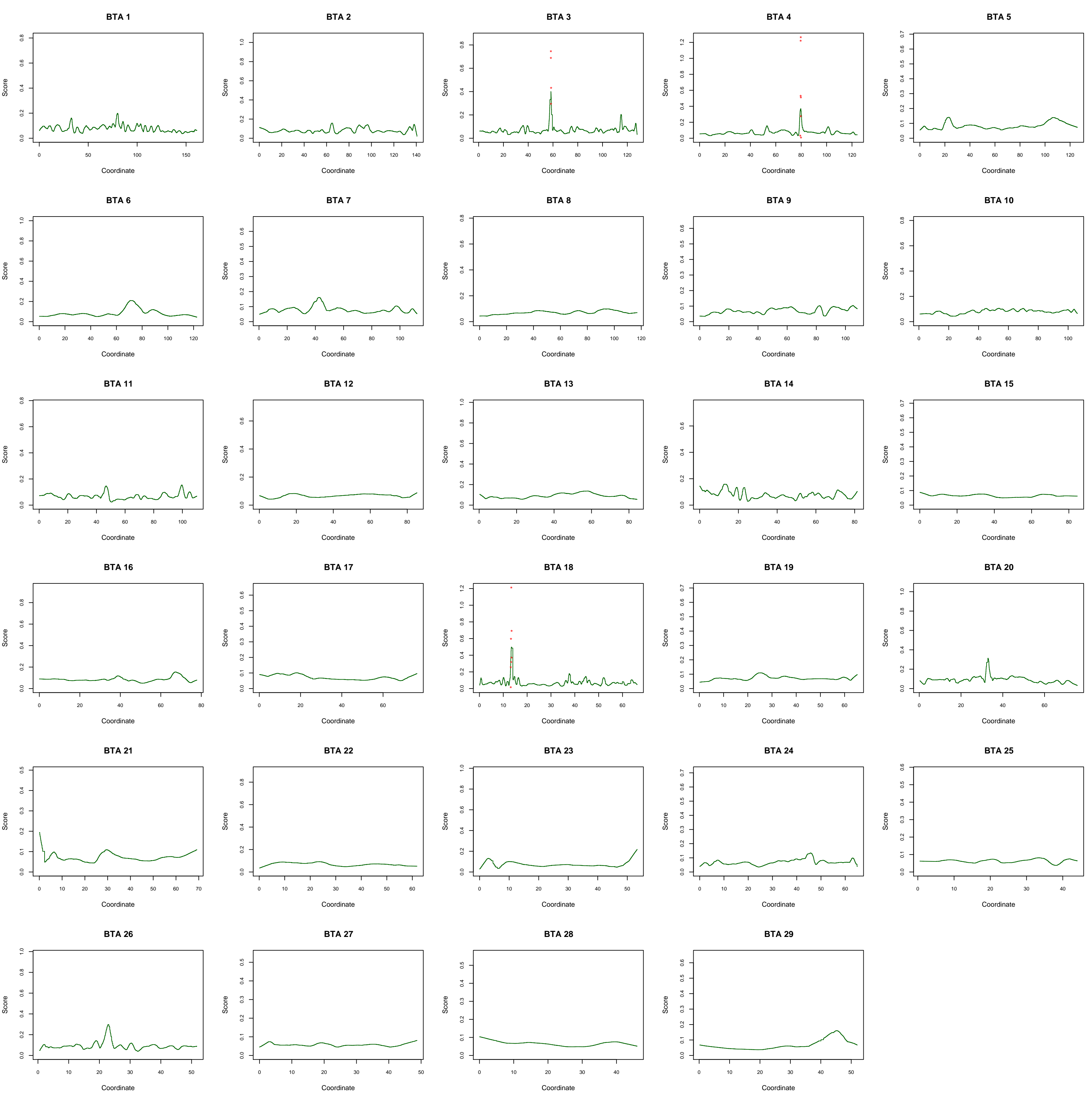

Supplement: Figure S5 — Whole genome map of regions under selection based on the FST within HOL. For each of the 29 bovine autosomes, the smoothed FST is plotted against the chromosomal position (green line). For significant positions (q-value<0.05), non smoothed SNP FST are indicated by a red star. (0.23 MB ZIP) [file pone.0006595.s008.zip › FigureS5.pdf]
